# Electrochemically induced *in vitro* focal hypoxia in human neurons

**DOI:** 10.3389/fcell.2022.968341

**Published:** 2022-09-28

**Authors:** Joseph J. Y. Wong, Balazs V. Varga, Ragnhildur Thóra Káradóttir, Elizabeth A. H. Hall

**Affiliations:** ^1^ Department of Chemical Engineering and Biotechnology, University of Cambridge, Cambridge, United Kingdom; ^2^ Wellcome—MRC Cambridge Stem Cell Institute, Cambridge, United Kingdom

**Keywords:** hypoxia, electrochemistry, microfluidic, human cortical neural progenitor, cortical neuron, axon, small vessel disease, lacunar infarct

## Abstract

Focalised hypoxia is widely prevalent in diseases such as stroke, cardiac arrest, and dementia. While in some cases hypoxia improves cellular functions, it mostly induces or exacerbates pathological changes. The lack of methodologies that can simulate focal acute hypoxia, in either animal or cell culture, impedes our understanding of the cellular consequences of hypoxia. To address this gap, an electrochemical localised oxygen scavenging system (eLOS), is reported, providing an innovative platform for spatiotemporal *in vitro* hypoxia modulation. The electrochemical system is modelled showing O_2_ flux patterns and localised O_2_ scavenging and hypoxia regions, as a function of distance from the electrode and surrounding flux barriers, allowing an effective focal hypoxia tool to be designed for *in vitro* cell culture study. O_2_ concentration is reduced in an electrochemically defined targeted area from normoxia to hypoxia in about 6 min depending on the O_2_-flux boundaries. As a result, a cell culture-well was designed, where localised O_2_ scavenging could be induced. The impact of localised hypoxia was demonstrated on human neural progenitor cells (hNPCs) and it was shown that miniature focal hypoxic insults can be induced, that evoke time-dependent HIF-1α transcription factor accumulation. This transcription is “patterned” across the culture according to the electrochemically induced spatiotemporal hypoxia gradient. A basic lacunar infarct model was also developed through the application of eLOS in a purpose designed microfluidic device. Miniature focal hypoxic insults were induced in cellular processes of fully oxygenated cell bodies, such as the axons of human cortical neurons. The results demonstrate experimentally that localised axonal hypoxic stress can lead to significant increase of neuronal death, despite the neurons remaining at normoxia. This suggests that focal hypoxic insult to axons alone is sufficient to impact surrounding neurons and may provide an *in vitro* model to study the impact of microinfarcts occurring in the deep cerebral white matter, as well as providing a promising tool for wider understanding of acute hypoxic insults with potential to uncover its pathophysiology in multiple diseases.

## Introduction

Cerebral small vessel diseases, such as white matter lesions and lacunar infarcts, are associated with increased risk of developing cognitive impairment and motor disturbances in neurodegenerative diseases (Prins and Scheltens, 2015; Alber et al., 2019; Ye et al., 2019; Liu et al., 2020; Snyder et al., 2021) and occur in the deep cerebral white matter (Smith et al., 2012; Martinez Sosa and Smith, 2017). Despite a clear relationship between cognitive decline and these small scale infarcts (2–20 mm in diameter) our understanding of the underlying mechanisms and clinical outcome of these asymptomatic “silent” infarcts is still lacking.

Oxygen homeostasis is crucial for cell function and depends on the control of blood circulation and cell metabolism. The effect of deprivation of oxygen in cells and activation of several signaling pathways is highly dependent on the duration and severity of hypoxia (Ferdinand and Roffe, 2016). While the detrimental effects of chronic hypoxia (long term hypoxic exposure) may be recoverable, acute hypoxia is significantly more harmful and induces necrosis, apoptosis, reactive oxidative species generation and inflammation (Saikumar and Venkatachalam, 2003; Sharp and Bernaudin, 2004; Imtiyaz et al., 2010; Hernansanz-Agustín et al., 2014).

In the human brain, the partial pressure of oxygen is about 35 mmHg (4.6%) decreasing with brain depth (Carreau et al., 2011). Permanent brain tissue damage can occur within minutes following a hypoxic event, followed by secondary damage to the surround tissue (penumbra). Cells and tissues activate adaptive responses that are primarily regulated by the constitutive cellular expression of Hypoxia Inducible Factor 1 alpha (HIF-1α) (Semenza, 2001). Hypoxia causes increased HIF-1α levels that activates cellular functions for both survival and cell death. In addition to the primary damage caused by cellular responses amid ischaemia, reperfusion accelerates the deleterious effect, commonly known as the secondary damage or the oxygen paradox (Daniele et al., 2021). Despite the consequences of focal acute hypoxia in small vessel diseases, knowledge of the temporal cellular mechanisms involved is still incomplete (Daniele et al., 2021). This is obstructed by the absence of appropriate experimental models to study the impact of acute focal hypoxia *in vitro*.

*In vitro* methods often utilise diffusional gas exchange (e.g., low oxygen incubators and hypoxia chambers), enzymatic oxygen scavengers (e.g., glucose oxidase with catalase (Li et al., 2016)) and HIF-1α degradation inhibiting chemicals (e.g., CoCl_2_ and DMOG (Anderson et al., 1994; Cummins et al., 2008)) which create homogeneous oxygen concentration across the entire culture (cf. 2–20 mm localised hypoxia in lacunar infarcts) but show slow (hours) diffusion and equilibration of oxygen (Allen et al., 2001). Chemically induced hypoxia targeting the HIF-1α pathway, only mimics the effect on the HIF-1α degradation mechanism, but not other O_2_-related pathways.

Pre-treated solution exchange with microfluidic devices (Piret et al., 2002; Thomas et al., 2011; Wang et al., 2014) reduces time for oxygen equilibration and avoids oxygen removing enzymes and chemicals causing unrelated side-effects in cells. However, these devices still limit differential spatial focal control of oxygen levels that would simulate the brain structure and the infarct (Brennan et al., 2014; Ando et al., 2017; Rexius-Hall et al., 2017). Some also require pumps for a constant flow of medium which affects the cell viability due to induction of shear stress (Wang C. J. et al., 2008; Herzog et al., 2021). A robust, rapid and focally targeted hypoxia platform, is required to study *in vitro* cellular mechanisms underlying focally induced hypoxia and to impact future therapeutic strategies.

In this work we investigate the principle of electrochemical Localised Oxygen Scavenging (eLOS) to apply focal acute hypoxia in the study of *in vitro* cell models. To achieve this goal, we investigate the oxygen gradient next to an electrode polarized at the oxygen reduction potential, and propose a design to induce the spatiotemporal hypoxia via electrochemical oxygen scavenging. The eLOS system is demonstrated in human neural progenitor cells (hNPCs), to show the spatiotemporal gradient of hypoxic response. The platform is further developed in a microchannel device to study a human cortical lacunar infarct model with pluripotent stem cell (PSC)-derived human cortical neurons.

## Materials and methods

### Materials and equipment

All chemicals were analytical grade. Different batches of reagents were prepared and compared throughout the study; no batch effects were observed. Phosphate buffer saline (PBS), potassium phosphate dibasic, potassium phosphate monobasic, potassium chloride, water for cell culture, borate buffer (pH 8.5), glycine, triton x 100, Mowiol solution, bisBenzimide H 33258, hydrogen peroxide, horseradish peroxidase (HRP) and dimethyloxalylglycine (DMOG) were supplied by Sigma Aldrich. DMEM/F-12 with GlutaMAX (with or without phenol red), neurobasal medium (with or without phenol red), GlutaMAX, sodium bicarbonate, beta-mercaptoethanol, N2 supplement (100x), B27 supplement (50x), B27 supplement without vitamin A (50x), insulin (human recombinant, zinc solution) and bovine serum albumin fraction V (7.5%) were obtained from Gibco. Animal-free blocking solution, and cleaved-caspase-3 (Asp175) antibody #9661 were obtained from Cell Signal Technology. 6F inhibitors were purchased from Selleckchem as listed in Varga et al., 2022. Poly-d-lysine 1 mg/ml in water was obtained from Millipore. Accutase and MitoSpy Green FM were obtained from BioLegend. Laminin used was obtained from Cultrex. German glass coverslips (ø:12 mm) were purchased from Bellco Glass Inc. Alexa Fluor 555-conjugated goat anti-rabbit polyclonal antibody, Amplex UltraRed were obtained from Thermo Fisher Scientific. Rabbit anti-HIF-1α monoclonal IgG antibody (1:200) (#ab51608) and Phalloidin-iFlour 488 Reagent—CytoPainter (Abcam #ab176753) are from Abcam. Chicken anti-TUBB3 (#NB100-1612) is from Novus Biological.

Electrochemical potential control and electrochemical data were obtained with a μStat 400 Bipotentiostat/Galvanostat from DropSens operating in the poteniostat mode. Scanning electron microscopy (SEM) was obtained on a SFEI Nova NanoSEM. Confocal microscopy used the TCS SP5 Leica Confocal Microscope. Microfabrication used the POLOS spin coater, 2.6-L Zepto Low Cost Plasma Laboratory Unit by Diener Plasma-Surface Technology, dataphysics OCA, DektaXT and the Karl Sus Contact Mask Aligner MJB-4. A Formlabs 3D printer was used for prototyping.

### Preparation of electrodes

The oxygen scavenging electrode was 5% platinum/graphite (Pt/C) kindly fabricated and provided by Alphasense, The Sensor Technology Company, Great Notley, Essex. Platinum was dispersed into ground-graphite with surfactant and mixed with PTFE, dried, then heated to remove water and surfactant and sintered near the melting point of PTFE in a proprietary fabrication process, then pressed into a Gore membrane (Ø: 14 mm). A copper wire was connected to the centre of the electrode. Details and images are given in Supplementary Section S1.

### Preparation of the clark-type oxygen electrode and oxygen electrode array

**FIGURE 1:**
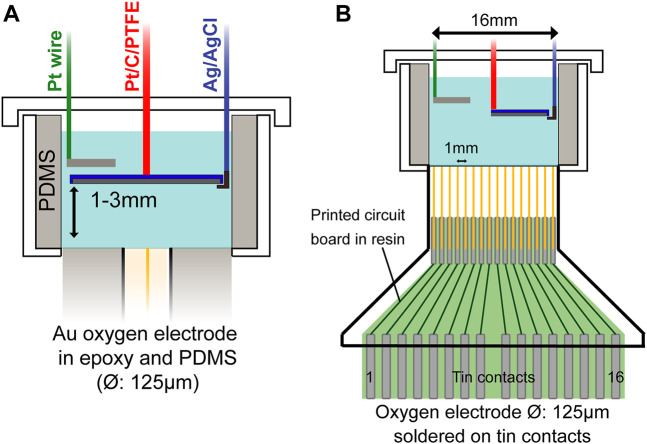
Schematic diagram of eLOSS set-up with Pt/C scavenging electrode and with Clark-like O_2_ measuring electrode(s) in base. **(A)** Scavenging electrode covering entire cell and **(B)** a ‘half’ electrode giving an oxygen gradient across the culture compartment for *in vitro* cell culture.

A Clark-type electrode and electrode array were used to measure oxygen concentration at the base of the electrochemical cell during oxygen scavenging (Figure 1). The single electrode (99.99+% purity gold wire Ø: 125µm, Goodfellow) was encased in epoxy resin of 4 mm diameter. The gold electrode 16 × 1 array, was arranged with each electrode positioned 1.0 mm apart across the 16 mm base of the cell-culturing compartment, for spatial mapping of the oxygen gradient. The array was formed by four 3D printed blocks (UV-curing clear resin), using a Formlabs 3D printer, that together encased the gold electrodes which were soldered on a custom printed circuit board (PCB). The construction detail is given in Supplementary Section S2. Characterisation of the electrode is presented in Supplementary Figure S1.

### Cell lines and culture

Human embryonic pluripotent stem cell-derived cortical neuroepithelial stem cells (cNESCs) were cultured and expanded from the stock (Sheffield 6, PMID: 28594288) (Varga et al., 2022). 1.5 ml of laminin (1.5 μg/cm^2^) and fibronectin (1 μg/cm^2^) containing PBS solution was added to the 6-well plates overnight before cNESCs were plated with a concentration of 50,000–100,000 cells/cm^2^. The cells were cultured in the 6F culture medium, composition detailed in (Varga et al., 2022).

Glass coverslips (Ø: 12 mm) were placed in 24-well plates and were cleaned in a nitrogen plasma cleaner for 10 min. The coverslips were coated with poly-D-lysine (3 μg/cm^2^) dissolved in borate buffer pH 8.5 for 1 h at 37°C, followed by laminin (1.5 μg/cm^2^) in PBS overnight at 37°C before cell attachment. Cells of 100,000/cm^2^ were plated on the coverslips and cultured in an incubator with 5% O_2_/7% CO_2_/N_2_ balance at 37°C. On the day before the hypoxic stress treatment, the culture medium was changed into FGF2 (10 ng/ml) included Neuron Differentiation Culture Medium (N2B27), which contains 1:1 ratio of DMEM-F12: Neurobasal medium, Glutamax (2 mM), Beta-mercaptoethanol (0.1 mM), N2 supplement (0.5x), B27 supplement (0.5x) and Insulin (10 μg/ml). This allows the cells to differentiate into hNPCs.

### Microchannel device fabrication

The microchannel device was a two-layer structure on a silica wafer (ø: 4”). The design and dimensions are given in Supplementary Figure S2. In brief, the silica wafer was cleaned with acetone and isopropanol, and dried with compressed gas before evaporating the solvents at 140°C. SU-8 2005 negative photoresist were spin-coated onto the clean silica wafer: 5 s at 500 rpm at 100 rpm/s acceleration, followed by 30 s at 3000 rpm at 300 rpm/s acceleration. The wafer was prebaked at 65°C (1 min) and 95 °C (2 min), followed by UV exposure (2 × 4s) and post-exposure baking at 65°C (1 min) and 95°C (3 min). Features were developed for 2 min in PGMEA before cleaning with isopropanol and hard baking at 150°C (15 min). The second layer followed a similar procedure. SU-8 2025 was deposited at a maximum spin-coating speed of 1700rpm. UV exposure was 2 doses of 10s. Baking times at 65°C and 95°C lasted for 3 min and 9 min, respectively, whist hard baking at 150 °C required 45 min. The wafer was characterized with a vertical profiler (Supplementary Figure S2C,D). A 3D-printed block was used to exclude PDMS (Sylgard 184) from the central compartment. After removal, a biopsy punch (Ø: 8 mm) and a scalpel were used to create the wells and trim the devices, which were bonded on glass slides (22 × 60 mm, Thermal Scientific Menzel #1.5) after activating by oxygen plasma for 40s (see section 3.7).

### Oxygen scavenging characterization in the eLOS

All experiments were conducted in bulk 0.1M KCl solution buffered with 40 mM phosphate buffer pH7.3 ± 0.1 (PBKCl) unless otherwise stated. Cyclic voltammetry, linear sweep voltammetry and chronoamperometry were used to study oxygen reduction and its adsorption on the electrode surface. The Anson equation was used to obtain charge due to oxygen reduction (Q_diff_) (Islam and Kant, 2011):

$$\begin{array}{c} Q_{diff}=2nFACD^{\frac{1}{2}}\pi^{-\frac{1}{2}}t^{\frac{1}{2}} \end{array}$$ (1)

where n is the number of electrons in the redox reaction, F is Faraday’s constant, A is the electrode area, C is concentration, D is diffusion coefficient and t is time.

A plot of Q vs. t^1/2^ was used to interpret the total charge, which includes Q_diff_, double layer charging, gas trapped in void electrode space, and adsorption (Q_ads_). Q_ads_ can be resolved from the projected intercept of the plot. Surface concentration of adsorbed species (Γ_0_) is calculated from Q_ads_ based on the following equation

$$\begin{array}{c} Q_{ads}={nFA\Gamma }_{0} \end{array}$$ (2)

In bulk oxygen-depleted experiments electrolyte was bubbled with nitrogen for 30 min before measurement began and then an over-pressure of N_2_ maintained above the electrolyte during measurements. BET analysis was conducted to study the electrode surface area and porosity.

A PDMS cell culturing well (Ø: 16 mm) was designed to accommodate the electrode system (Figure 1A). The details of construction are given in Supplementary Section S3. The scavenging electrode was placed at ∼1.0mm and ∼1.5 mm distance from the bottom of the well. The oxygen measuring electrode was fixed at the PDMS base, polarized at -0.5 V for 3 min to stabilize the current before switching on oxygen scavenger for 30 min. The O_2_ measuring electrode was positioned at the centre (0 mm) and well edge (6 mm radial distance from the centre), for investigating the uniformity of oxygen concentration across the base. Alternatively, the electrode array was embedded in the base of the well (Figure 1B) to enable oxygen concentration to be mapped with greater resolution across the diameter cross-section. Measurements were performed in air, N_2_ and in 5% O_2_ concentration with a 5% O_2_/N_2_ balance premixed gas cylinder.

### Electrochemical simulation

A finite difference model (MATLAB 2021a) was used to simulate the change of oxygen concentration under the oxygen scavenging system and verify the measurements taken by the oxygen electrode. The Nernst-Plank equation was solved to predict and map the oxygen concentration change from ambient 5% or 20.9% O_2_ partial pressure, under the influence of a diffusion-controlled oxygen scavenging electrode system. This gives:

$$\begin{array}{c} \frac{\partial C}{\partial t}=-D\nabla C-\frac{zF}{RT}DC\nabla \phi +Cv \end{array}$$ (3)

where z is the number of electrons involved in the reduction of oxygen, ϕ is the electrode potential, R is the universal gas constant, T is the temperature and v is the velocity of the fluid and ∇ is the gradient. C, D and t are defined as above.

Based on the absence of convection, high background electrolyte content and uncharged oxygen molecules, the equation is simplified to a diffusion problem. As the system dimensions are significantly larger than the steps and by symmetry, the problem can be assumed and discretized as a 2D Cartesian grid, giving:

$$\begin{array}{c} \frac{\partial C}{\partial t}=D\left[\frac{\partial^{2}C}{\partial x^{2}}+\frac{\partial^{2}C}{\partial y^{2}}\right] \end{array}$$ (4)

where x and y are horizontal and vertical in the Cartesian grid respectively. Implementing the finite difference method, this simplifies the equation to:

$$\begin{array}{c} \frac{C_{\left(i,j,k+1\right)}-C_{\left(i,j,k\right)}}{dt}=D\left[\frac{C_{\left(i+1,j,k\right)}{+C}_{\left(i-1,j,k\right)}-{2C}_{\left(i,j,k\right)}}{dx^{2}}+\frac{C_{\left(i,j+1,k\right)}+C_{\left(i,j-1,k\right)}-{2C}_{\left(i,j,k\right)}}{dy^{2}}\right] \end{array}$$ (5)

where i, j and k are indexes for x, y and t, respectively.

The model simulates the PDMS well-based system described above. Both a full circular electrode and a half circle electrode were considered at heights at 1–3 mm from the base. The base is modelled with different materials, having a mix of O_2_ diffusion properties including a PDMS, glass, epoxy or polystyrene. Glass, epoxy and polystyrene boundaries are assumed to be no flux boundaries due to the low diffusion coefficient of oxygen in these materials. The input values for the model are given in Table 1.

**TABLE 1:** Parameters used for simulation.

Parameter	Physical interpretation	Value	Units	Reference
X	Total length in horizontal direction	10	Mm	-
dx	X steps	0.067	Mm	-
Y	Total length in vertical direction	5 (no flux base)/8 (PDMS base)	mm	-
dy	Y steps	0.033 (no flux base)/0.053 (PDMS base)	mm	-
T	Total time	1800	sec	-
dt	Time step	0.075	sec	-
[O_2_]_ambient_	Oxygen partial pressure in percentage in ambient	5/20.9	%	-
D_solution_	Diffusion coefficient of oxygen in solution	2.0 × 10^–5^	cm^2^ s^−1^	
D_PDMS_	Diffusion coefficient of oxygen in PDMS	4.0 × 10^–5^	cm^2^ s^−1^	
D_epoxy_	Diffusion coefficient of oxygen in epoxy	4.9 × 10^–7^	cm^2^ s^−1^	Chowdhury et al. (2016)
D_polystyrene_	Diffusion coefficient of oxygen in polystyrene	1.7 × 10^–7^	cm^2^ s^−1^	Ghosal and Freeman (1994)
X_pdms	Thickness of the PDMS wall in *x* direction	2	mm	-
Y_pdms	Thickness of the PDMS base	3	mm	-
X_elec	length of the electrode	7 (half)/14 (full)	mm	-
Y_E_pos	Position of the scavenging electrode from the base	1–3	mm	-
**Boundary**		**Boundary conditions**			
Centre of well		Symmetric boundary condition			
Air-solution interface		Dirichlet boundary condition (Fixed concentration boundary, [O_2_] = [O_2_]_ambient_)			
Glass, polystyrene, epoxy interface		Neumann boundary condition (No flux boundary)			
Electrode polarized to reduce oxygen		Dirichlet boundary condition (Fixed concentration boundary, [O_2_] = 0)			

### pH and H_2_O_2_ generation

Changes in pH and H_2_O_2_ concentration were measured after polarizing the electrode at the oxygen reduction potential for 0 min, 30 min and 3 h. The measurements were conducted in PBKCl and neural maintenance medium, with or without catalase (≥12.5U/mL). H_2_O_2_ concentration was measured with Amplex UltraRed assay as described in Supplementary Section S3 and Supplementary Figure S3, S4. pH was measured with a Jenway 3510 pH meter.

The effect of H_2_O_2_ at different concentrations was also studied on hNPCs cultures. Cells were cultured in their basic culture medium with H_2_O_2_ supplemented, with or without 1.25 μg/ml of catalase. H_2_O_2_ at final concentrations of 1 µM, 10 µM, and 100 µM were added to the culture medium immediately before adding to cells. Staurosporine (SRP) 100nM, a broad-spectrum protein kinase inhibitor was added to the media as the positive control. Cells were all cultured for 4 h in 5% O_2_.

### Hypoxia induction by eLOS and characterization

The PDMS well-container was cleaned in a nitrogen plasma cleaner (10 min) before adding an hNPCs-attached glass coverslip to the base of the container (creating a no-O_2_ flux boundary) and placing in a CO_2_ incubator. FGF2 added N2B27(-), N2B27 without phenol red, was used over the hypoxic treatment. The scavenging electrode was positioned at ∼1.5 mm away from the cells and polarized at -0.7 V for electrochemical induction of hypoxic stress for 1 h, 3 h, and 6 h. The hypoxia positive control was cultured in the presence of the drug DMOG (250 µM) for 6 h. Other culturing conditions were the same as above. Acute hypoxia was conducted at time intervals of 20 mins, 40 mins and 60 min.

To spatially localise the hypoxia, the scavenging-electrode was shaped by cutting. Circular and half circular electrodes were compared placed above the cells (Figure 1B). The electrode was polarized at -0.7 V and the outcome followed for 3 h. The focusing of the electrochemical removal of oxygen on one half of the culture dish, using the half-circle electrode was compared with the full-circle. Hypoxic responses were analysed based on the cells’ respective spatial positions. Fixed cells were imaged as tiles along the horizontal axis. Mean HIF-1α intensity within the cell nucleus was used for analysis.

### Focal acute hypoxia in the microchannel device by eLOS

Clean microchannel devices were coated with PDL and laminin according to the procedure described above. After 14 days in the N2B27, immature neurons were detached by 15 min of Accutase incubation and centrifuged at 400x g for 3 min 100,000 resuspended cells, typically in 10µL, were seeded next to the channels in the side chambers, protected by a 90 mm petri-dish and humidified with sterile water in a 35 mm petri-dish. Higher solution height was maintained in the central chamber in the first week to keep a gentle outflow, deterring neurons from migrating through the barrier channels. After 3 weeks of culturing in N2B27, neurons extended axons across the barrier channels and formed networks in the central chamber.

The cultures were then subjected to hypoxic stress in N2B27(-). The Pt/C O_2_-scavenging electrode (6 mm × 7 mm) was placed in the central chamber at 2 mm height. In acute hypoxia experiments, oxygen was scavenged for 25 and 35 min, corresponding to 15 and 25 min at focal hypoxia in the central chamber, respectively. For bulk hypoxia cells were incubated in 0.2% O_2_/5% CO_2_ in N_2_ for 6 h (3 h equilibration + 3 h bulk hypoxia).

An immediate N2B27 media change was done at the end of the hypoxia time period with 18 h post hypoxia incubation in 5% O_2_ according to the normal levels of O_2_ in cells. The negative control was kept in 5% O_2_ throughout. All samples were fixed after the experiment and were imaged by confocal microscopy following the procedures in Supplementary Section S4.

### Immunofluorescent staining

Cells were fixed with 4% paraformaldehyde (PFA) for 10 min and rinsed twice with PBS. Fixed samples were first blocked with Animal Free Blocker and Diluent (AFB) containing 0.3 M glycine and TritonX0.1 for 1 h in room temperature.

The primary antibody for the hypoxia induction study targeted HIF-1α. It used recombinant rabbit anti-HIF-1α monoclonal IgG antibody, ab51608 from Abcam with dissociation constant, K_D_ = 2.24 × 10^−10^ M. 5 μg/ml rabbit anti-HIF-1α monoclonal IgG antibody (1:200) (Abcam #ab51608) (Storti et al., 2014) was diluted in AFB. Primary antibody staining lasted for 2 h at room temperature. Slides were then rinsed three times in PBS with TritonX0.1 for 5 min each. They were incubated in AFB containing 2 μg/ml Alexa Fluor 555-conjugated goat anti-rabbit polyclonal antibody for 1 h at room temperature in the dark. Slides were rinsed 3 times in PBS with TritonX0.1.

All slides were stained with bisBenzimide H33258 for 1 min before mounting and then dried in the dark overnight. Z-stacks were taken at ×20 magnification with the confocal microscope (Leica Microsystems, TCS SP5 Microscope).

Neurons in microchannel devices were stained with TUBB3 and bisBenzimide H33258. Primary antibody staining used chicken anti-TUBB3 (#NB100-1612) (1:1000) in AFB for 16 h at 4 °C, followed by secondary antibody anti-chicken 488 (1:500) for 2 h.

The apoptotic response study had most of its steps similar to the hypoxia induction study. 1 μg/ml rabbit polyclonal anti-caspase-3, active (cleaved) form primary antibody (1:200) (Merck #AB3623) was used as the primary antibody and secondary antibody used was same as above. Actin filament staining was performed after the washes of the secondary antibody staining. Phalloidin-iFlour 488 Reagent—CytoPainter (Abcam #ab176753) stock (50x) in PBS +1% BSA were prepared in advance and stored in the fridge until use. It was diluted to 1x working solution shortly prior to use and added to the coverslips. Samples were incubated for 20 min in room temperature, followed by 3 times rinsing with PBS with TritonX0.1 before nucleus staining.

### Data analysis

All experiments were conducted with positive and negative controls in parallel (Table 2). All experiments had 3 independent replicates (*n* = 3) unless stated otherwise. A minimum of six images were taken randomly per experiment per replica. Each immunofluorescence image contains a minimum of 100 cells and had their respective signal intensities quantified and positive cells counted by the Volocity^®^ software. Background threshold was selected based on the Huang’s thresholding (Huang and Wang, 1995). Results were plotted in graphs presenting the mean percentage ±standard deviation. Minimal intensity thresholds were selected as the signal intensity at mean +3 S.D. of negative control, while maximum intensity was selected as the mode of the respective positive control. Frequency distribution in acute hypoxia was analyzed with K-S test. Apoptotic cells were quantified by their respective caspase-3 positive signal around the cell nucleus. All results were compared statistically by paired *t*-test. The Holm-Bonferroni test was conducted on the resultant p values to avoid type I errors and p values less than 0.05 were considered statistically significant. Chromatin condensation in microchannel devices had >200 cells counted per replicate (n = 4). Dunnett’s test was conducted and p values less than 0.05 were considered statistically significant.

**TABLE 2:** Experimental design.

Experimental design	Experiment	Conditions/parallel experiments	Replicates
Characterisation of Pt/C	*Oxygen adsorption*	In ambient	3
	*Oxygen adsorption*	In nitrogen	3
	*BET*	In nitrogen	3
	*Tafel plot*	In nitrogen	3
	*Oxygen concentration change*	In nitrogen	3
eLOS hypoxia 6 images randomly taken with minimum 100 cells each; minimum 1,000 cells per replicate	*Hypoxic response*	Negative control	3
	*Hypoxic response*	Time conditions	3 each
	*Hypoxic response*	Positive control (DMOG 250 µM)	3
	*Acute hypoxia*	Negative control	3
	*Acute hypoxia*	Time conditions	3
	*Focal hypoxia*	Negative control	3
	*Focal hypoxia*	Position conditions	3
	*Focal hypoxia*	Positive control	3
Apoptosis study	*pH change*	Different solutions	3 each
	*H*_*2*_ *O*_*2*_ *generation*	Different solutions	3 each
	*hNPC apoptosis*	Negative control	3
	*hNPC apoptosis*	H_2_O_2_ concentrations	3 each
	*hNPC apoptosis*	Positive control (Staurosporine 100 nM)	3
Neuron focal hypoxia	*Hypoxia at microchannel device*	Positional conditions	3
	*Cortical neuron model*	Negative control	4
	*Cortical neuron model*	Focal hypoxia conditions	4 each
	*Cortical neuron model*	Bulk hypoxia	4

## Results and discussion

### Electrode O_2_ scavenging characterization

The key proposal of the research reported herein is that spatially localized hypoxia can be induced by electrochemical reduction and removal of the oxygen near an electrode placed at the target hypoxia site. Electrochemical reduction of oxygen is a pH dependent 2- or 4-electron reaction that depletes dissolved O_2_ from the diffusion layer next to an electrode and leads to H_2_O_2_ or OH^−^ (H_2_O) respectively. The competition between the 2- and 4- electron pathways is determined by pH and electrode catalyst. Pt strongly promotes the 4e route, with density functional theory (DFT) calculations suggesting that Pt catalyses the O–O bond cleavage and O–H bond formation in reduction of oxygen rather than forming H_2_O_2 (Pang *et. al.* 2020)*,*_ The proposed configuration for electrochemical oxygen scavenging is shown in Figure 1A with a gas permeable membrane Pt/C electrode placed above the cell culture in close proximity to the cells. The PTFE/Pt/C membrane electrode was chosen as scavenger electrode to fulfil the design criteria of oxygen scavenging and promote the 4-electron reduction to H_2_O. In terms of O_2_ scavenging normalised to geometric area, the Pt/C electrode has a high O_2_ reduction current density of ∼20 pmol/mm^2^/s (over an order of magnitude larger than that on gold: -0.9 pmol/mm^2^/s, Supplementary Figure S5). The favourable Pt/C O_2_ reduction can be attributed in part to the high surface area that is created with O_2_ pre-adsorbed in proximity to the catalytic Pt particles. The irregular topography of the heterogeneous sintered PTFE/Pt/C electrode (Supplementary Figure S6B–F) allows further advantageous increase in reactive sites for the oxygen reduction reaction.

**FIGURE 2:**
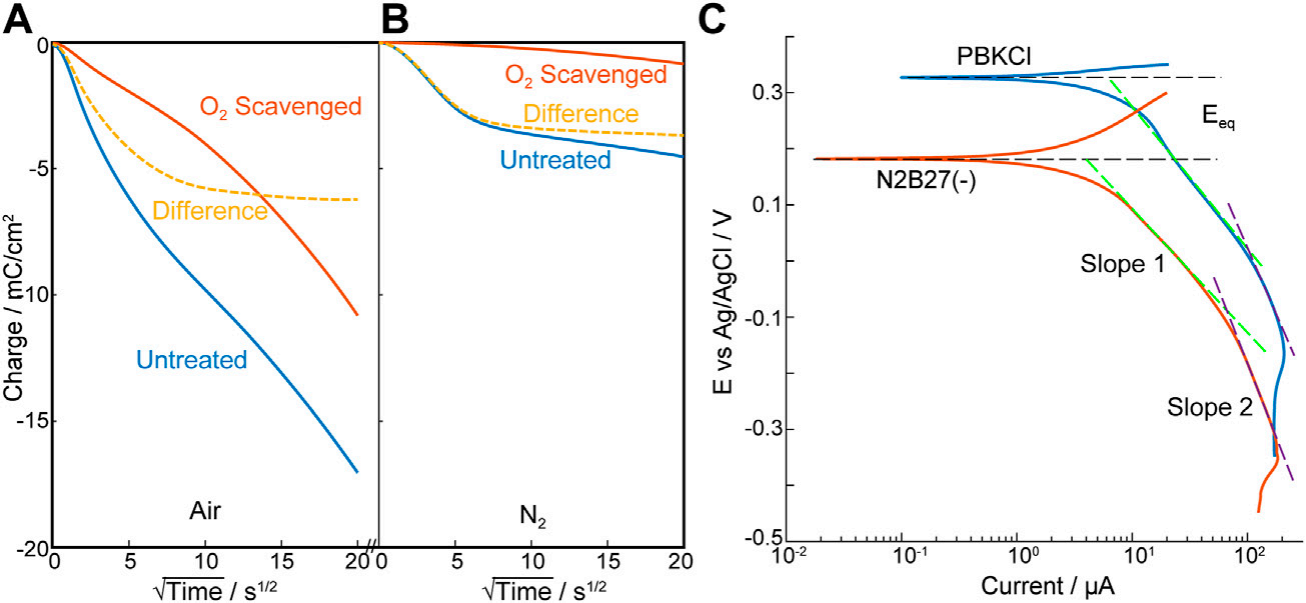
Characterization of the scavenging electrode—Pt/C. **(A,B)** Anson plots of oxygen adsorption and scavenging analysis of Pt/C electrode, polarized at -0.7 V vs. Ag/AgCl electrode in PBKCl measured in air **(A)** and nitrogen **(B)**. O_2_ scavenged Pt/C electrode (Red); Untreated Pt/C electrode (Blue); Difference (Yellow dotted). **(C)**. Tafel plot of Pt/C electrode under 20.9% O_2_ in PBKCl and N2B27(–) respectively (*n* = 3).

In BET surface analysis (Supplementary Figure S6G, H) the sintered 5% Pt/C electrode was seen as non-porous (or microporous), with a monolayer N_2_ adsorption of 48 ± 7 cm^2^/mg, giving a BET area of ∼330 cm^2^ per electrode. Chronoamperometry on the Pt/C electrodes produced an initial decrease in cathodic current due to reduction of pre-adsorbed oxygen, before reaching steady state diffusion-controlled oxygen reduction (Supplementary Figure S7). Comparing the response from air-pre-equilibrated and post-O_2_-scavenged electrodes, 80.3 ± 2.2 nmol of oxygen was estimated to be adsorbed on the electrode (Figure 2A). However, even after long equilibration in N_2_ an estimated 40.9 ± 4.1 nmol O_2_ is still available for electrochemical reduction (Figure 2B). Assuming the latter is a monolayer of strongly adsorbed oxygen and taking the cross-sectional area of O_2_ as 0.147 nm^2^ (Ismail, 1992), this corresponds to 10.83% of the measured BET area. This is consistent with reduction occurring at the Pt sites (5%) with some limited scavenging from immediately adjacent C sites. The increase in adsorbed O_2_ after equilibration in air is consistent with further more weakly bound O_2_ on the electrode (and thus able to be purged in N_2_).

In the oxygen reduction reaction, Pt containing electrodes have typically shown two Tafel slopes (∼60mV/Dec and ∼120mV/Dec) (Perez et al., 1998; Schmidt et al., 2001; Ge et al., 2015) attributed to the pivot between O and OH or OOH adsorption in the reaction pathway (Wang J. X. et al., 2008). In contrast, Tafel slopes (Figure 2C) on this Pt/C/PTFE electrode suggest much lower catalytic activity (232 ± 10mV/Dec and 492 ± 37mV/Dec). This is reminiscent of graphite fibre/epoxy electrodes reported by Nacamulli and Gileadi (Nacamulli and Gileadi, 1983) and may be associated with the dominance of graphite and the low Pt loading (5%). However, while increasing the Pt loading might increase the catalytic activity it also increases background electrochemical hydrolysis of water and, importantly, hydrogen microbubble evolution, that could reduce the viability of the cells. The wider cathodic potential window achieved at low Pt loading, without causing H_2_ evolution was an advantage in accommodating small solution-dependent shifts observed in the O_2_ reduction potential (e.g., in PBKCl at -0.3 V vs. Ag/AgCl) and N2B27(-) culture media at -0.35 V vs. Ag/AgCl) (Supplementary Figure S8). This 5%Pt electrode was thus chosen for this feasibility study.

### Electrochemical oxygen-reduction hypoxia model development

The electroreduction process for O_2_ can be used to measure oxygen concentration such as in the Clark O_2_ electrode, or deployed for scavenging the interfering dissolved oxygen in biosensors, demonstrated by Hahn et al. (1982; Bacon and Hall, 1999). It is investigated here as a method to induce local hypoxia. Spatial focal resolution of hypoxia can be modulated through the shape and size of the electrode and its positioning relative to the target hypoxia site in a cell culture. In the case of lacunar infarcts, the required resolution for a viable experimental *in vitro* model is 2–20 mm, thus electrodes were chosen for this study with dimensions between 8 and 16 mm cross section.

**FIGURE 3:**
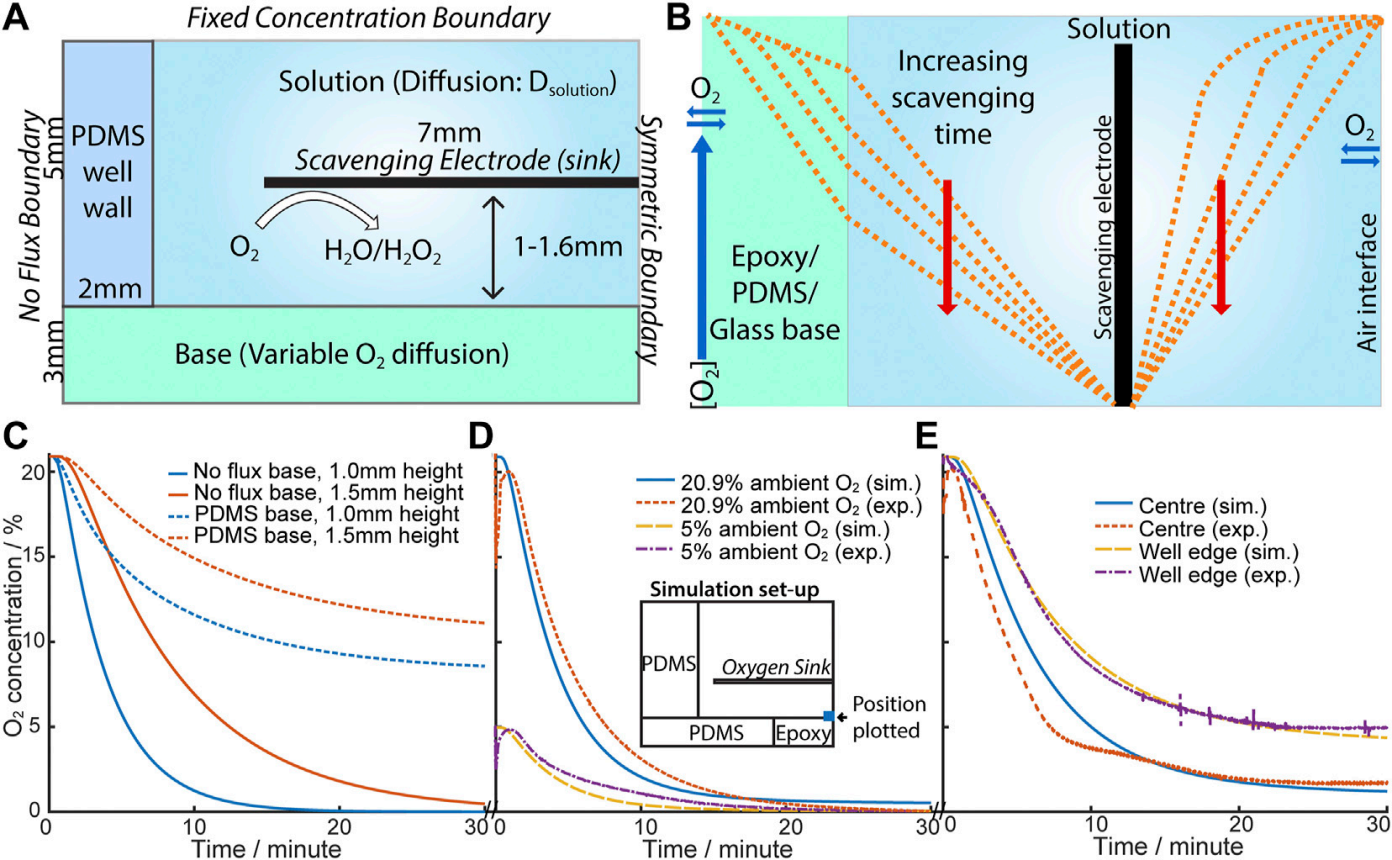
**(A)** Schematic diagram of the simulation of eLOS. **(B)** Schematic illustration of the oxygen concentration change when the oxygen scavenger is switched on (oxygen sink). **(C)** Plot of simulation results with no flux (epoxy/glass) base (solid line) and flux (PDMS) base (dotted line); 1.0 mm (blue), 1.5 mm (red). **(D)** Comparison of exp. and sim. [O_2_] in ambient [O_2_] at 20.9% and 5%. Inset: schematic of simulation set-up used. Scavenger is at 1.1 mm above base. **(E)** Comparison of exp. and sim. [O_2_] across the base. Scavenger is at 1.5 mm above base.

A mathematical model of a scavenging electrode was first built based on a culture-well with dimensions as shown in Figure 1A and having an oxygen scavenging electrode placed above the cell culture, which is permeable to oxygen. Figure 3A shows this as a cross section through half the proposed culture-well, with the symmetric boundary lying through the centre of the culture well (with an O_2_ measuring electrode placed at the centre in the base of the well). The well sides and base provide O_2_ flux or no-flux boundaries depending on the material used (see Table 1 for the input parameters and boundary conditions). Figure 3B shows a diagram of the expected development of O_2_ concentration gradients with time, after the scavenging electrode has been switched on; this anticipates that O_2_ scavenging will compete with O_2_-diffusion (if any) across the well-base and well-walls. If the well-base allows O_2_ flux (e.g., through PDMS, Figure 3C, dotted line), the scavenging electrode at 1 mm height above the base, competes with the influx of O_2_ across the base and is only able to reduce [O_2_], measured at the centre of the well, to circa 50% of ambient. In contrast, the [O_2_] drops towards zero at the no flux well-base (Figure 3C, solid line), and the time required depends on the distance from the scavenging electrode. It is intuitive that distance dependence from the site of ischaemic infarct is also featured in the impact of localised lacunar infarcts.

Based on these predictions an experimental culture-well model was built to characterise the eLOS, and having an oxygen measuring electrode in the well base, to monitor oxygen scavenging. The configuration of the experimental cell is shown in Figure 3D, insert; the experimental model contains flux and no flux interfaces. Although flux of O_2_ across the well-base is shown not to be desirable for the final hypoxia model, an initial experimental proof of model system, used an oxygen permeable PDMS structure, which allowed good flexibility in changing and configuring the cell, for characterisation of the performance of the scavenging electrode. The input values for the theoretical model could accommodate these variations and indeed allowed for a hybrid diffusion barrier along the base.

For example, the initial experimental model had a 4 mm diameter epoxy resin encased Au-O_2_-measuring electrode inserted into the PDMS base; the 4 mm epoxy introduced a no-flux boundary area in the centre of the well-base. For a scavenging electrode placed at ∼1 mm above the base, the model (Figure 3D) shows a good fit to a scavenging electrode at 1.1 mm from the base and reaches a steady state at ≤2% ambient. With this hybrid diffusion base to the well, hypoxia (defined as < 1.5–2% O_2_ (Semenza et al., 1997)) from 20.9% ambient O_2_ is produced in ∼15 min compared with ∼6 min from 5% ambient O_2_. hNPCs and cortical neurons are cultured in 5% O_2_ so this 6 min timeframe is consistent with that expected in permanent cellular damage which can occur within minutes.

Similar conclusions are drawn for electrodes placed at ∼1.5 mm above the base, where good fits to the model are also shown with the measuring electrode placed at different positions across the well-base (Figure 3E). In all cases the greater distance between the scavenger electrode and the well-base, the higher the resultant steady state O_2_ concentration; of particular note is the change in steady-state oxygen seen after scavenging, moving across the base, with the electrode monitoring ∼9% of ambient oxygen at the centre of the well and higher levels (∼27% of ambient) measured at the O_2_ monitoring electrode placed near the edge of the well, close to the PDMS well-wall. O_2_ flux is not inhibited across this side wall, so the result is intuitive. This also illustrates the potential to design 3-D cell-culture structures with different flux boundaries to simulate different organ and scenario models.

### Mapping of dissolved oxygen in cell-culture medium following eLOS

**FIGURE 4:**
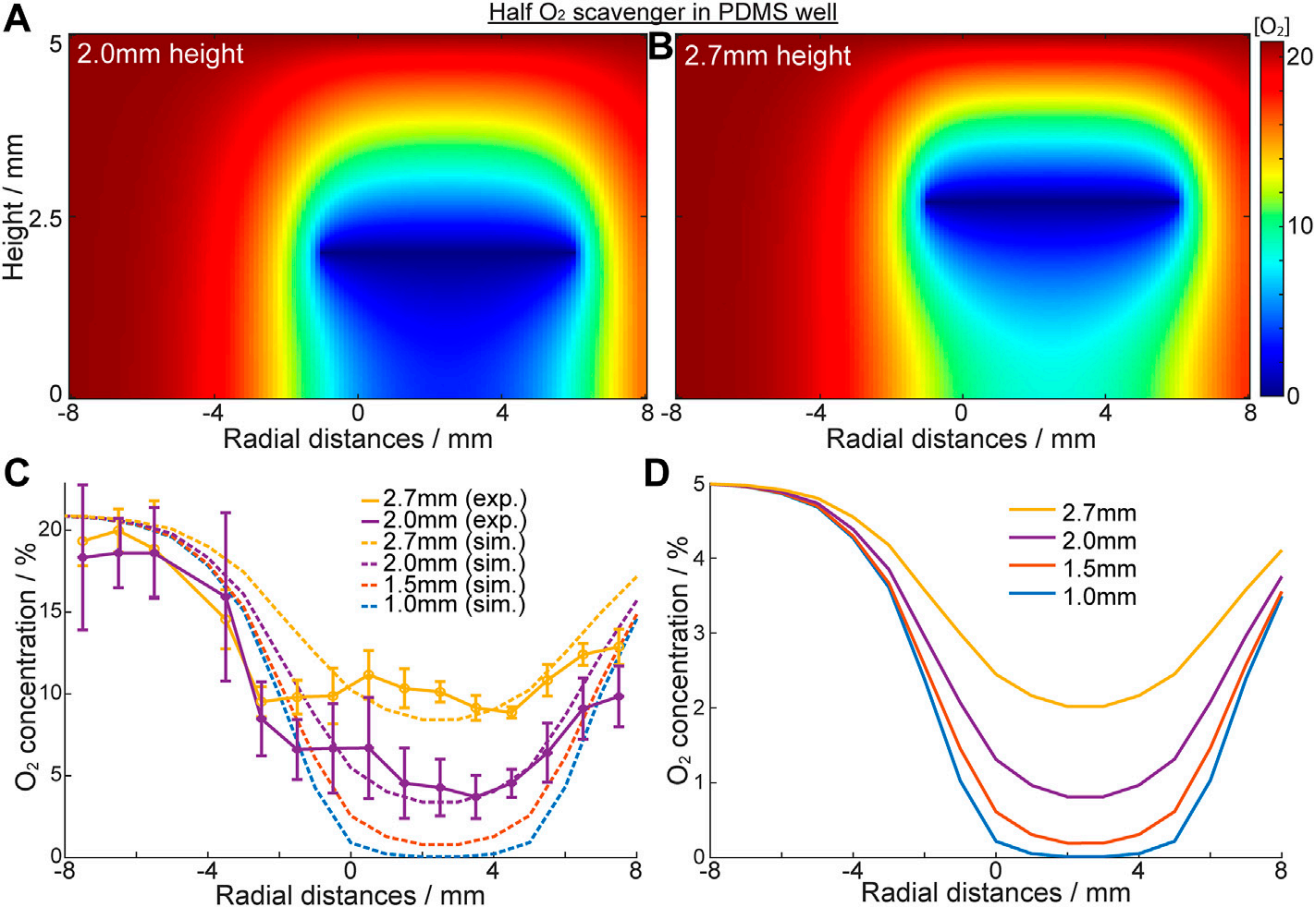
Characterisation of oxygen concentration “patterning” with “half” scavenging electrode at -1mm–6 mm. **(A,B)** Heat map of oxygen concentration of the solution inside the PDMS well with a no flux base in [O _2_]_ambient_ = 20.9%. Oxygen scavenging electrode is a half circle, positioned at 2.0 mm **(A)** and 2.7 mm **(B)** height. **(C)** Oxygen concentration across the well base when electrode is placed at different heights. Solid line: experimental results; dotted line: simulation results. **(D)** Simulated [O_2_] in the same system when [O_2_]_ambient_ = 5% (cell culture ambient).

As established above, residual O_2_ concentration increased with radial distance from the centre under the scavenging electrode to the outer edge (6 mm), where diffusion of oxygen through the PDMS wall competes with the scavenging. The spatial resolution of oxygen concentrations across the well-base during oxygen scavenging can be further explored for an electrode shaped to selectively cover only a target area in a cell culture (Figure 1B). Figures 4A,B show the [O_2_] heat-map for this “half-electrode” positioned at different heights from a no-flux well-base, as predicted from the model. For electrodes at 2.0mm and 2.7 mm above the base, the model shows that at 20.9% ambient O_2_, hypoxia (<1.5% O_2_) extends only ∼1.2 mm from the scavenging electrode. In these conditions (Figure 4C) only ∼85% and 50% of the oxygen can be removed at the well base, respectively for electrodes at 2.0 and 2.7 mm height. However, at 5% ambient O_2_ (the normal condition for human cell cultures used in this study) hypoxia is induced even at the base of the well at 2.0 mm (Figure 4D). This is consistent with the data above for the full electrode. It is also observed that the area of low oxygen maps the electrode shape and dimensions, suggesting the ability to spatially resolve the area of hypoxia with dimensions appropriate to lacunar infarcts.

To further authenticate the models, an O_2_ monitoring electrode array was built into the well-base, across the diameter of the well, and was used to confirm the eLOS model results above (Supplementary Figure S9). The experimental data (Figure 4C) shows a reasonable fit with the simulated models at both 2.0mm and 2.7 mm heights. The model also predicts that for heights below 1.5 mm hypoxia will be achieved at the well-base under the scavenging electrode even at 20.9% ambient. From these data we ascertain that spatial resolution of hypoxia is related to the ambient oxygen concentration, the electrode shape and distance from the hypoxia source. In design of an *in vitro* eLOS culture well, it is also clear that the well-base should be a no flux oxygen boundary and the side walls could be a flux or no-flux oxygen boundary (depending on the study parameters required) to achieve the resolution and control for local hypoxia.

### Electrochemically induced hypoxia in hNPCs

**FIGURE 5:**
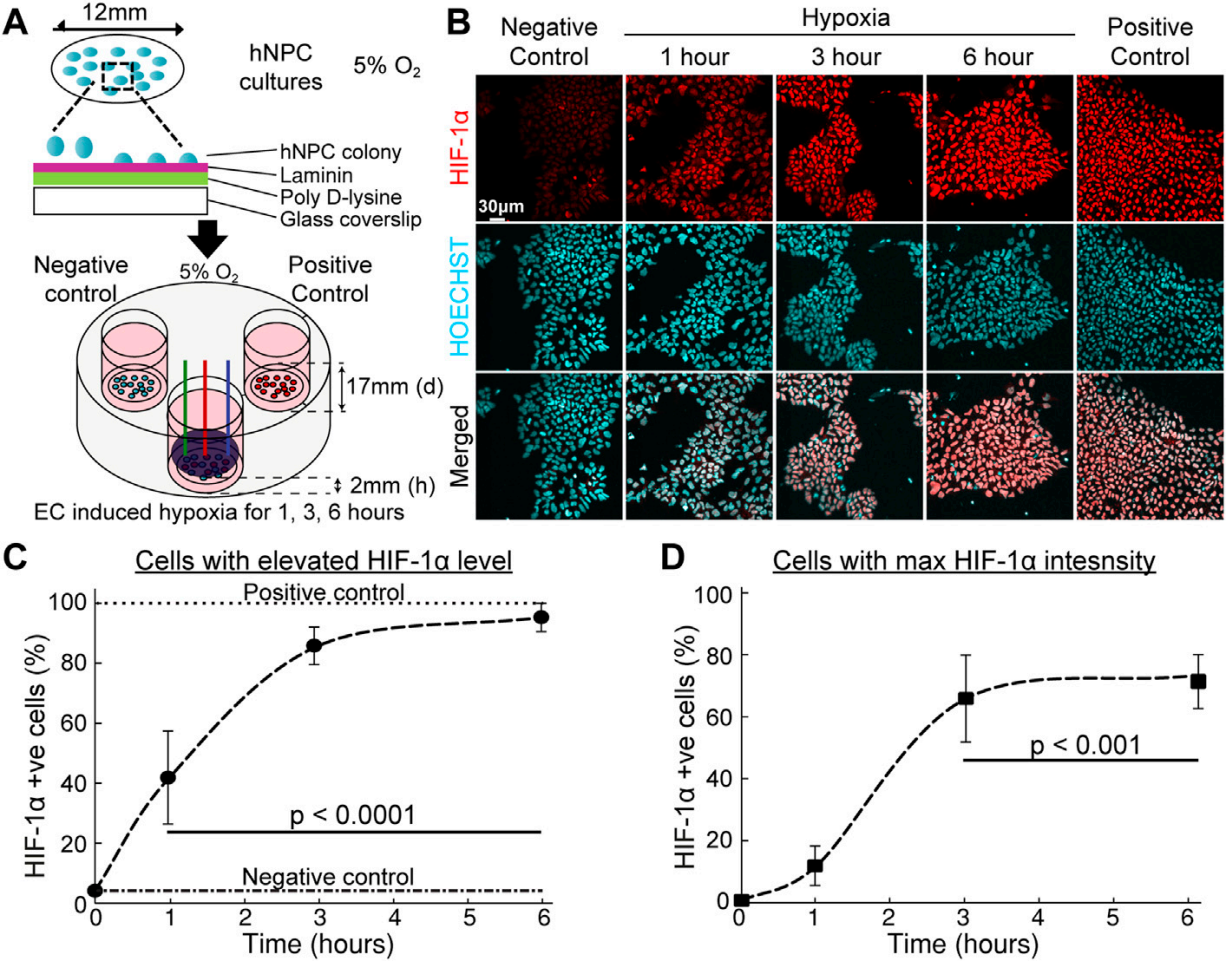
Hypoxic response of hNPCs in electrochemical incubator. **(A)** Schematic procedure of the preparation of hNPCs and scavenging system set-up. **(B)** Representative confocal images of cell cultures with immunofluorescent staining on HIF-1α (red) and nucleus (blue). **(C,D)** Quantitative analysis of HIF-1α response under long duration of hypoxic insult. (*n* = 3, error bars indicate standard deviation). **(C)** Percentage of cells with HIF-1α fluorescent intensity above base HIF-1α intensity. **(D)** Percentage of cells attaining maximum HIF-1α fluorescence intensity, compared with HIF-1α intensity in the presence of DMOG, see Supplementary Figure S10.

Based on this foundation, the eLOS platform was tested to explore its ability to control hypoxia in cultured cells. hNPCs can be cultured at physiological oxygen levels (3–5%) and are more resilient to hypoxia (Figure 5A) (Varga et al., 2022). Cells cultured in 5% O_2_ were exposed to electrochemically induced hypoxia for 1–6 h. The cellular response to hypoxia were quantified by the levels of HIF-1α in the cells by immunofluorescent labelling (Figure 5B).

Absence of oxygen inhibits the hydroxylation dependent degradation of HIF-1α which will lead to increased level of HIF-1α under conditions of low oxygen availability. The number of cells with elevated HIF-1α levels positively correlated with duration of eLOS hypoxia (Figures 5C,D): the cellular HIF-1α positive cell ratio changed from 42% of the cells at 1 h to a plateau of 95% at 6 h (Figure 5C). The level of eLOS-induced HIF-1α was comparable with chemical stabilisation by DMOG. The proportion of cells reaching maximum HIF-1α intensity showed a sigmoidal increase with time (Figure 5D, 12% at 1h, 66% at 3h and 73% at 6 h of hypoxia) and no necrotic cells were detected, marked by pyknotic nuclei. These results show that cells impacted by electrochemically induced hypoxia show a canonical HIF-1α hypoxia response; this infers that oxygen levels can be reduced to create a localized hypoxia gradient around the cells by electrochemical O_2_ scavenging.

**FIGURE 6:**
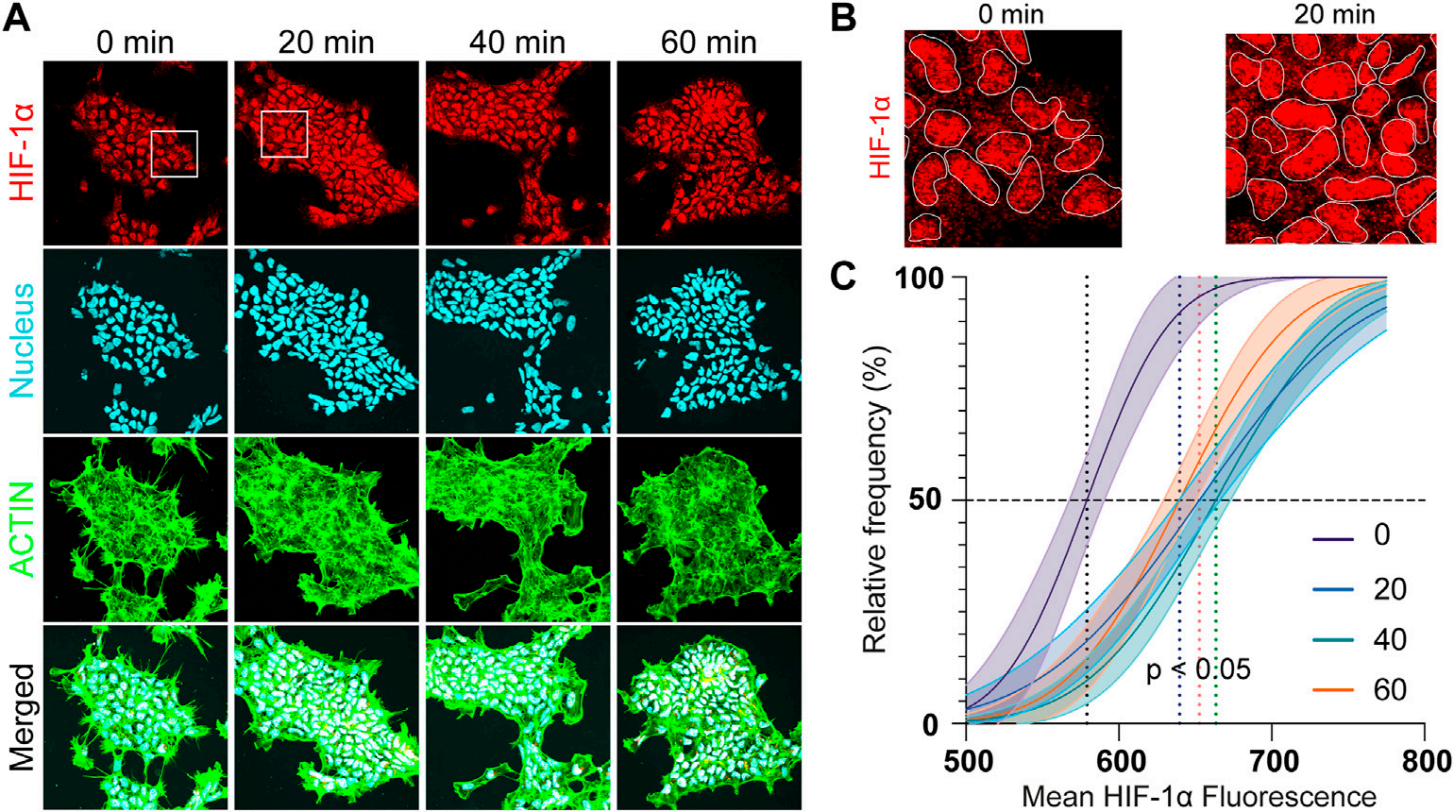
Acute hypoxia of hNPCs. **(A)** Representative confocal images of hNPCs in acute hypoxia: HIF-1α (red); nucleus (cyan); actin (green). **(B)** Zoom in of the confocal images for comparison. **(C)** Frequency distribution analysis of the mean HIF-1α intensity in the cells under different hypoxia durations.

To observe the immediate effects of acute hypoxia, the HIF-1α intensity change of hNPCs was investigated within the first hour of scavenging for an electrode covering the whole culture. An upregulation of the HIF-1α signal was detected within 20 min of hypoxic insult (*p* = 0.005) (Figure 6A) and is clearly observed in the nucleus of the cells (Figure 6B). Mean HIF-1α intensity is shifted from 598 ± 21A.U. in the negative control towards ≥650A.U. (*p* < 0.05) under focal hypoxia (Figure 6C), demonstrating the capability of the eLOS to induce a rapid hypoxic response as suggested by the characterization results reported above, faster than that observed with DMOG (250 µM) treatment (Supplementary Figure S10).

### Potential side products of eLOS oxygen reduction

To ensure that the results obtained above could be attributed to hypoxia only, the products of electrochemical oxygen reduction like H_2_O_2_ and OH^−^ (pH) were investigated. pH can affect most cellular processes such as metabolism and growth. Peroxide is known to lead to apoptosis at high concentrations (Schmidt et al., 2001; Beyhan et al., 2015). The impact of the O_2_ reduction products on the culture medium were studied from 30 min and up to 3 h where further changes were not observed. As discussed in section 3.1, the Pt catalysed reduction of oxygen is expected to favour the 4-electron route to H_2_O, rather than the 2-electron route to H_2_O_2_. Nevertheless, the oxygen electroreduction was followed under cell-culture conditions to assess the potential for H_2_O_2_ as a minor side product.

In buffered media solutions, as would be used in these *in vitro* cell based experiments, no pH changes were detected during eLOS under all conditions tested (Table 3). This is as expected for the 4-electron reduction in buffered solution. Low concentration of H_2_O_2_ was detected in a simple buffer solution - PBKCl medium (800 nM H_2_O_2_ was estimated from the AUR fluorometric method Supplementary Figure S3) but still below biologically critical H_2_O_2_ concentrations. With commonly used DMEM/F-12 cell-culture medium, only a trace of H_2_O_2_ was detected between 30 min and 3 h eLOS (estimated to be ≤ 400 nM H_2_O_2_ in both cases, close to the formal detection limit Supplementary Figure S4). Gülden *et. al.* (2010) estimate a median cytotoxic dose of 870nM/10^7^ C6 glioma cells, with cells able to remove H_2_O_2_ with time below this concentration, due to the normal cell scavenging mechanisms.

**TABLE 3:** pH and H_2_O_2_ concentration under eLOS oxygen scavenging.

Scavenging conditions (n = 3)	Measurand	Time (mins)		
		0	30	180
PBKCl PBKCl	*pH* *[H_2_O_2_]*	7.3 ± 0.1 Negative	7.3 ± 0.1 0.8 ± 0.3 µM	7.3 ± 0.1 0.8 ± 0.3 µM
PBKCl with catalase PBKCl with catalase	*pH* *[H_2_O_2_]*	7.3 ± 0.1 Negative	7.3 ± 0.1 Negative	7.2 ± 0.1 Negative
DMEM/F-12 DMEM/F-12	*pH* *[H_2_O_2_]*	7.6 ± 0.1 Negative	7.6 ± 0.1 0.8 ± 0.3 µM	7.6 ± 0.1 Trace amount
DMEM/F-12 with catalase DMEM/F-12 with catalase	*pH* *[H_2_O_2_]*	7.4 ± 0.1 Negative	7.4 ± 0.1 Negative	7.4 ± 0.1 Negative

However, adding 12.5 U/mL catalase as present in the culture medium formula, is expected to totally remove any H_2_O_2_ generated. Indeed, no H_2_O_2_ was detected in any samples under these conditions. It should also be noted that diffusion of H_2_O_2_ from the electrode is not likely to extend any further than the distances seen in the O_2_ depletion zones in Figures 3A,B and the concentration will diminish with distance from the electrode, which suggests that only a subtoxic amount of H_2_O_2_ will be present at any point away from the electrode surface and that all H_2_O_2_ will be consumed by catalase. Furthermore, the highest H_2_O_2_ concentrations would be expected immediately after the onset of oxygen scavenging when the oxygen concentration is highest; the rate of oxygen reduction reaction, interpreted from the current, reduces with time to a steady state (Figure 3). Thus, the rate of production of H_2_O_2_ (if any) will be highest at the start, yet it remained below the detection limit in the presence of catalase. The data indicate that apoptosis is unlikely to result from the products of oxygen reduction in the proposed 4 electron electroreduction of oxygen.

**FIGURE 7:**
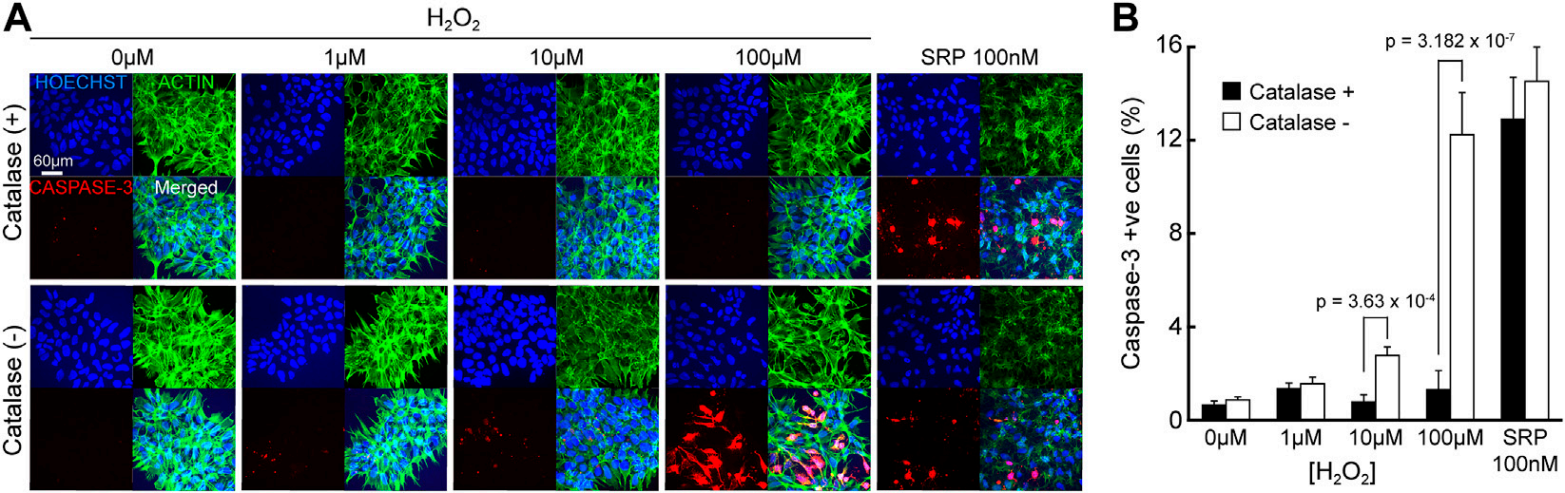
eLOSS does not induce pH or apoptotic stress. **(A)** Confocal images of hNPCs after 4-h culture in culture medium containing H_2_O_2_ with or without catalase: Nucleus (blue), Caspase-3 (red), Actin (green). **(B)** Quantitative analysis of percentage of cells with caspase-3 activation under different H_2_O_2_ concentrations. SRP: staurosporine.

Nevertheless, these results should be considered in context; the impact of H_2_O_2_ on hNPCs morphology and apoptosis, is indicated by the presence of caspase-3, a key mediating protein that activates cell death. Immunofluorescent images (Figure 7A) showed hNPC nuclei are intact at all concentrations of H_2_O_2_ in catalase (+) culture medium and no sign of caspase-3 activation was seen, even up to 100 µM H_2_O_2_. This is more that 3 orders of magnitude higher than the H_2_O_2_ detected from the electroreduction of O_2_, in the absence of catalase. In catalase (-) medium, caspase-3 was activated when H_2_O_2_ reached 10 µM but morphological changes like actin degradation, nuclear condensation and fragmentation were only observed at 100 µM H_2_O_2_ (Figure 7B). Thus, these effects only occur at concentration higher than H_2_O_2_ accumulation during eLOS even in the absence of catalase. These results strongly indicate that eLOSS only results in hypoxic stress to the cells.

### Tailor made hypoxia gradient in single culture

**FIGURE 8:**
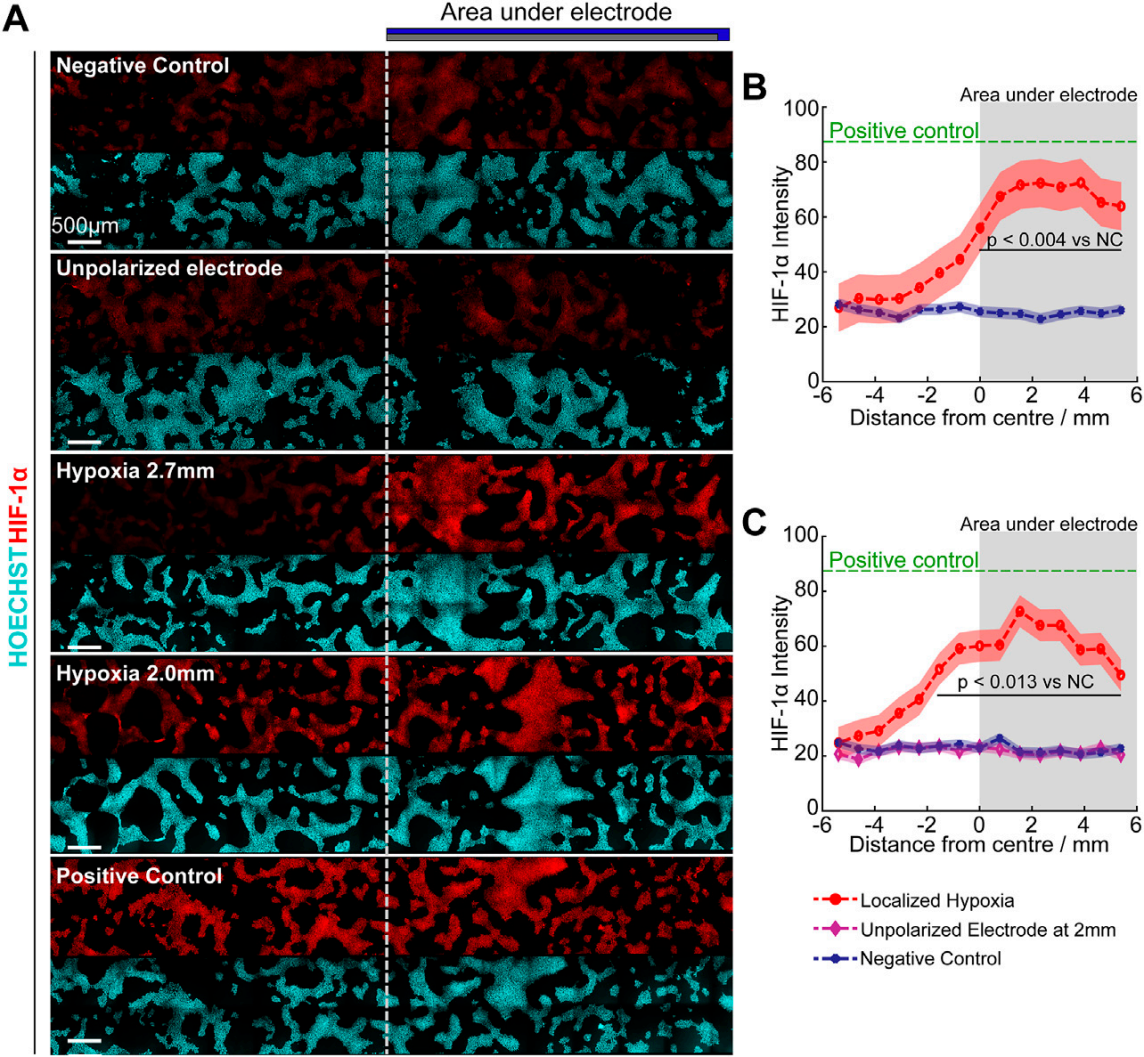
Focal hypoxia in a well. **(A)** Confocal images of cultures under different hypoxia conditions. Dotted line indicates the border of the electrode. **(B,C)** Quantitative analysis of mean HIF-1α intensity (curve) ± standard deviation (shaded region) across the base under 2.7 mm **(B)** and 2.0 mm **(C)** height of scavenging electrode, analysed with Tukey multiple comparison. Green: Positive control (250 µM DMOG); Red: Scavenging electrode polarized; Purple: Scavenging electrode unpolarized; Blue: Negative control. Grey shaded area indicates regions under oxygen scavenging electrode.

To take a further step towards localised eLOS hypoxia within a cell culture without physical separation of cells, the “half-electrode” eLOS system described and characterized above with a no-flux base was transferred to cell culture. A target-area of cells in a bulk culture volume was defined by the shape of an electrode placed above the culture. The “half-electrode” format described in Figure 1B was chosen to define the target shape and the effect of eLOS was characterized by the gradient of HIF-1α intensity in immunofluorescent images of hNPCs after hypoxia. This correlated with the cells’ position on the well base, with respect to the electrode (Figure 8A) and with the local O_2_ spatial calibration in this format (Figure 4).

Quantifying the HIF-1α intensity, verified that maximum hypoxic response was observed underneath the oxygen scavenging electrode (shaded region, Figures 8B,C) and reduced hypoxic stress was apparent at the edges due to the influx of oxygen from the air-water interface and across the PDMS-electrolyte interface. For the electrode positioned at 2.0 mm above the cells, the increased HIF-1α levels “leaked” slightly beyond the edges of the electrode shadow into the uncovered area. In contrast at 2.7 mm above the cells, only the area under the electrode showed statistically significant increase in HIF-1α levels.

Unexpectedly, the maximum HIF-1α intensity in both cases were similar despite having a lower oxygen concentration beneath the electrode at 2.0 mm above the cells (Figure 4D). However, this is consistent with the activation of proline 402 in the hypoxia recognition element of HIF-1α at circa 15 mmHg (2%) O_2_ (Figure 4D predicts 2% O_2_ for the 2.7 mm eLOS), whereas proline 564 is activated at circa 4 mmHg (0.5%) O_2_ (Figure 4D predicts 0.8% O_2_ for the 2.0 mm eLOS), but resulting in a similar accumulation rate for HIF-1α of the hNPCs (Chan et al., 2005). In the negative control and unpolarized electrode culture, HIF-1α intensity remained low and uniform across the cells, proving the HIF-1α readout was not caused by restriction of oxygen diffusion due to the physical presence of the scavenging electrode. This result provides a good basis to anticipate that further experimental models of localised hypoxia could yield new perspectives on the impact of an acute hypoxic shock in small scaled infarcts.

### Focal infarct model

**FIGURE 9:**
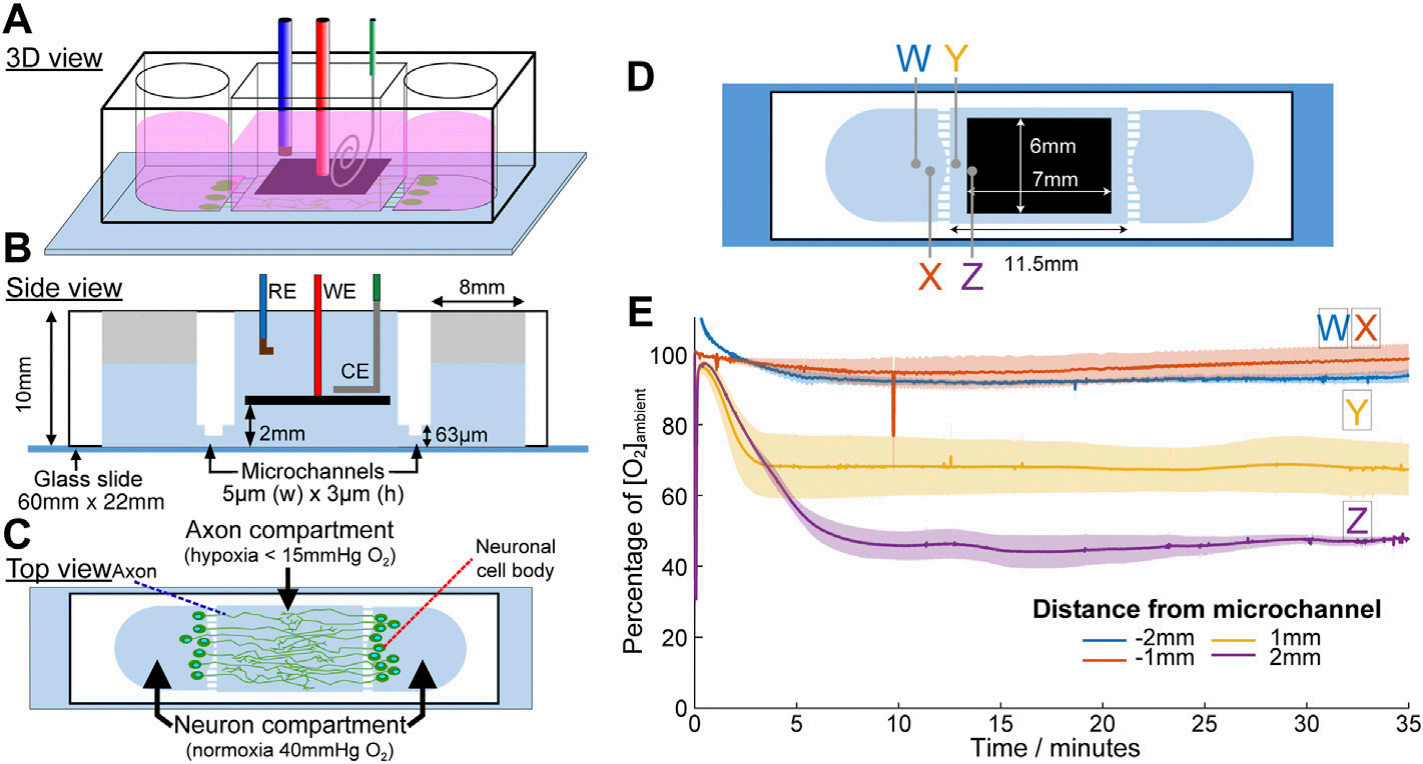
Microchannel device designed for the application of acute focal hypoxia in the human cortical brain model. Schematic diagrams for the device at 3D view **(A)**, side view **(B)** and top view **(C)**. Oxygen is only scavenged from the middle chamber where only axons are allowed to grow into. **(D)** Positions of the oxygen concentration measured were indicated by crosses in the respective colours. **(E)** Plot of oxygen concentration at different positions under eLOS, measured by a gold electrode.

Based on the results demonstrating acute focal hypoxia modelling capability, eLOS was applied in a basic lacunar infarct model. Lacunar infarcts appear in the white matter of the brain around the axons of neurons, millimetres away from the cell body of the neurons. A compartmentalised cell culture system was designed to miniaturise and confine hypoxia to the axons, as commonly observed in small vessel diseases (Figures 9A–C). Microchannels of 5 µm (h) x 3 µm (w) (profiled in Supplementary Figure S2) were designed to connect the neuron side chambers with a central chamber such that only the axons can grow into it. The scavenging electrode was positioned at 2 mm above the base of the central chamber and steady state oxygen concentration was achieved in 5 min (Figures 9D,E). In the central chamber, the lowest level of oxygen (∼55% decrease) occurred as expected, in the area furthest from the microchannels (position Z in the diagram), followed by 30% decrease at 1 mm from the microchannel (position Y). These contrast with the measurements in the side chambers where mirror positions (position W, X, respectively) remain similar to the ambience, irrespective of scavenging in the central chamber. These results demonstrate a highly efficient oxygen scavenging for focusing hypoxia in the central chamber, while leaving the side chambers in normoxia.

**FIGURE 10:**
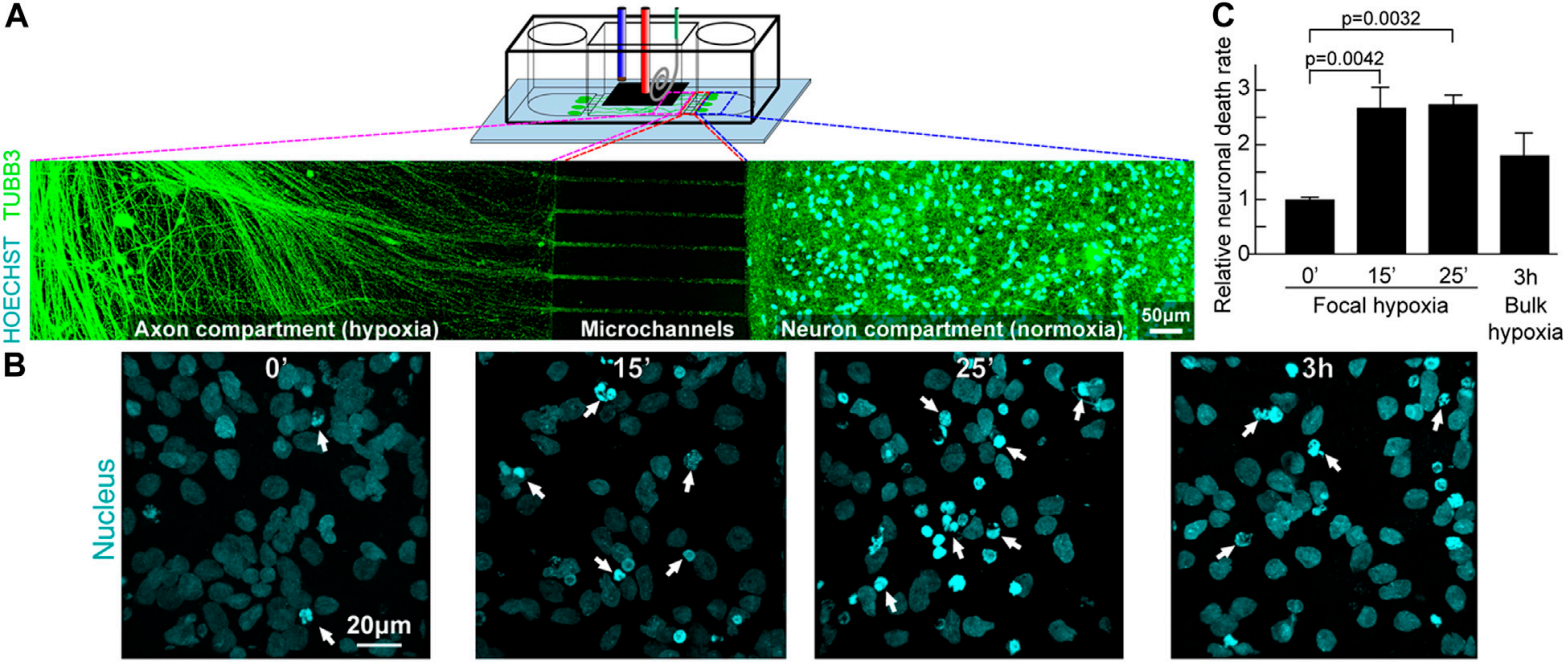
Focal hypoxia in a human cortical neuron microchannel model. **(A)** Schematic presentation of the microchannel model. Representative confocal tile image of neurons growing across the microchannels. Cyan: cell nucleus; green: axons. **(B)** Representative confocal image of cell nucleus (cyan) at the end chambers and axons in the central chamber after hypoxic insults. **(C)** Quantitative analysis of cells with chromatin condensation (*n* = 4).

Building upon the characterization results, the focal infarct model was developed from a PSC-derived human cortical neuron culture. Neurons were prepared and seeded into the side chambers; they had cell bodies restricted to those chambers by the microchannel dimensions (Figure 10A). Meanwhile the axons (stained green with TUBB3 in Figure 10A) could grow freely through the microchannels into the central chamber. To simulate a transient hypoxic shock, axons that had grown into the central chamber were subjected to 15 min and 25 min acute hypoxia, followed by reperfusion to normoxia for 18 h. As seen in Figure 10B, most neurons that are not been exposed to hypoxia had spherical or ovoid nucleus, commonly seen in most cell types. When the cells were exposed to hypoxia, significantly more cells had a different nuclear morphology, showing nuclear fragmentations and chromatin condensations that are associated with cell death. Quantifying the neuronal death rate in the side chambers, that had not been directly exposed to hypoxia, except through the connection of their axons (Figure 10C), the neurons showed significant 2.68 ± 0.38-fold (*p* = 0.004) and 2.74 ± 0.17-fold (*p* = 0.003) increase in chromatin condensation, respectively for 15 and 25 min axon hypoxia (normalised against the negative control with 10% cell death occurring normally after the cell seeding). It is also observed experimentally that despite the significant increase in neuronal death after acute hypoxia and reoxygenation, some neurons remain intact. As many axons extended only in the cell body compartment, this may correlated with the neurons which did not grow processes through the microchannels into the central chamber and thus these axons were able to escape focal hypoxia.

In contrast, 3h bulk ambient diffusion-based hypoxia followed by 18-h reoxygenation, exerted on both the central and side chambers, so that both neuron cell bodies and axons were in hypoxia, showed only 1.80 ± 0.41 fold (*p* = 0.175) increase in chromatin condensation, despite the hypoxic condition lasting much longer. These observations support a previous report that reducing oxygen concentration abruptly leads to more permanent neurological damage (Ferdinand and Roffe, 2016). The current study is the first to demonstrate experimentally that localised axonal hypoxic stress can lead to significant increase of neuronal death. The rate of oxygen change (abrupt vs. gradual) is critical to cell fate, and reinforces the case that incubator-dependent hypoxia models provide different information to that applicable to acute hypoxia studies. However, this is just the beginning of a new methodology in the study of focal hypoxia and further investigations can now emerge to elucidate the signaling pathways involved.

## Conclusion

This study demonstrated a novel electrochemical methodology for modelling *in vitro* acute focal hypoxia on human cortical neurons. Adjusting the vertical height, size and position of an electrode above a cell culture and controlling oxygen concentration through electrochemical oxygen scavenging, produces focal hypoxia that is spatiotemporally relevant to simulation of lacunar infarcts and neuronal damage. The methodology does not require additional chemicals or pumps and the [O_2_] can be reduced locally to hypoxia in less than 6 and 15 min at 40 and 159 mmHg ambience, respectively. This compares with 3 h in gas exchange-based hypoxia.

The potential for this methodology is demonstrated in the elevated HIF-1α levels positively correlated with duration and position of eLOS hypoxia in hNPCs. However, one of the most significant findings of this study with its novel hypoxia methodology, is its successful adoption in focal acute hypoxia in a human cortical neuron model of lacunar infarct. Using a microfluidic design providing spatial separation of neurons from their axons, a focal acute hypoxic insult on axons translated into significantly more chromatin condensation and nuclear fragmentation in connected human cortical neurons, that were cultured at normoxia, than in diffusion-based bulk hypoxia involving both the axons and neurons.

There may be significance from this finding in the study of neurodegenerative diseases. Historically, the main focus of research has been on neurons in the brain’s gray matter. Despite that, in Alzheimer’s disease white matter lesions occur before the onset of clinical symptoms and their accumulation correlates with the symptom severity. White matter is located at the distal end of the brain’s circulatory supply and this makes it relatively more susceptible than grey matter to changes in oxygen, glucose concentration and blood pressure. It consists mainly of axons, surrounded by myelin, produced by oligodendrocytes. Myelin integrity and axonal health are key to white matter health and microglia (situated in the white matter) are affected by vascular, axonal or myelin damage caused by micro hypoxic insults, which may perpetuate white matter lesions.

We believe that this eLOS platform overcomes many hurdles to study acute focal hypoxia in vitro cell culture. eLOS can improve spatiotemporal control of hypoxia in timescales of acute and chronic hypoxia (reduction rate and duration), and by more accurately modelling diseases spatiotemporally. Although this proof of principle, presents a small vessel diseases lacunar infarct model, this versatile system can be adapted for studying tumor microenvironment, myocardial infarction and other ischaemic systems *in vitro* with an advantage of live imaging of the underlying heterotypic interactions and their pathological mechanisms that will impact therapeutic strategies.

This exciting and potentially significant result opens the door for eLOS to be used in studying the signalling pathways and transcription factors activated in focal acute hypoxia and investigate focal hypoxia-related mechanisms in tissue culture and 3D organelles that will impact therapeutic strategies.

## Data Availability

The original contributions presented in the study are included in the article/Supplementary Material, further inquiries can be directed to the corresponding author.
