# Corrigendum: Electrochemically induced *in vitro* focal hypoxia in human neurons

**DOI:** 10.3389/fcell.2022.1118466

**Published:** 2023-01-05

**Authors:** Joseph J. Y. Wong, Balazs V. Varga, Ragnhildur Thóra Káradóttir, Elizabeth A. H. Hall

**Affiliations:** ^1^ Department of Chemical Engineering and Biotechnology, University of Cambridge, Cambridge, United Kingdom; ^2^ Wellcome—MRC Cambridge Stem Cell Institute, Cambridge, United Kingdom

**Keywords:** hypoxia, electrochemistry, microfluidic, human cortical neural progenitor, cortical neuron, axon, small vessel disease, lacunar infarct

In the published article, there was an error in Table 2 as published. This was due to a formatting error during publication causing rows to become misplaced in the final printed copy and subscript information to be lost.

**TABLE 2:** Experimental design.

Experimental design	Experiment	Conditions/parallel experiments	Replicates
Characterisation of Pt/C	*Oxygen adsorption*	In ambient	3
	*Oxygen adsorption*	In nitrogen	3
	*BET*	In nitrogen	3
	*Tafel plot*	In nitrogen	3
	*Oxygen concentration change*	In nitrogen	3
eLOS hypoxia 6 images randomly taken with minimum 100 cells each; minimum 1,000 cells per replicate	*Hypoxic response*	Negative control	3
	*Hypoxic response*	Time conditions	3 each
	*Hypoxic response*	Positive control (DMOG 250 µM)	3
	*Acute hypoxia*	Negative control	3
	*Acute hypoxia*	Time conditions	3
	*Focal hypoxia*	Negative control	3
	*Focal hypoxia*	Position conditions	3
	*Focal hypoxia*	Positive control	3
Apoptosis study	*pH change*	Different solutions	3 each
	*H*_*2*_ *O*_*2*_ *generation*	Different solutions	3 each
	*hNPC apoptosis*	Negative control	3
	*hNPC apoptosis*	H_2_O_2_ concentrations	3 each
	*hNPC apoptosis*	Positive control (Staurosporine 100 nM)	3
Neuron focal hypoxia	*Hypoxia at microchannel device*	Positional conditions	3
	*Cortical neuron model*	Negative control	4
	*Cortical neuron model*	Focal hypoxia conditions	4 each
	*Cortical neuron model*	Bulk hypoxia	4

The corrected Table 2 and its caption Experimental design appear below.

In the published article, there was an error in Table 3 as published. This was due to a formatting error during publication causing rows to become misplaced in the final printed copy.

**TABLE 3:** pH and H_2_O_2_ concentration under eLOS oxygen scavenging.

Scavenging conditions (n = 3)	Measurand	Time (mins)		
		0	30	180
PBKCl PBKCl	*pH* *[H_2_O_2_]*	7.3 ± 0.1 Negative	7.3 ± 0.1 0.8 ± 0.3 µM	7.3 ± 0.1 0.8 ± 0.3 µM
PBKCl with catalase PBKCl with catalase	*pH* *[H_2_O_2_]*	7.3 ± 0.1 Negative	7.3 ± 0.1 Negative	7.2 ± 0.1 Negative
DMEM/F-12 DMEM/F-12	*pH* *[H_2_O_2_]*	7.6 ± 0.1 Negative	7.6 ± 0.1 0.8 ± 0.3 µM	7.6 ± 0.1 Trace amount
DMEM/F-12 with catalase DMEM/F-12 with catalase	*pH* *[H_2_O_2_]*	7.4 ± 0.1 Negative	7.4 ± 0.1 Negative	7.4 ± 0.1 Negative

The corrected Table 3 and its caption pH and H_2_O_2_ concentration under eLOS oxygen scavenging appear below.

Error in Table carried over to the index figure.

In the published article, there was an error in Index figure as published. This arose as a carry-over of the error in the formatting of the table that the publishers used as index figure A corrected index figure appears below corresponding to Figure 10 in the manuscript.

**FIGURE 10:**
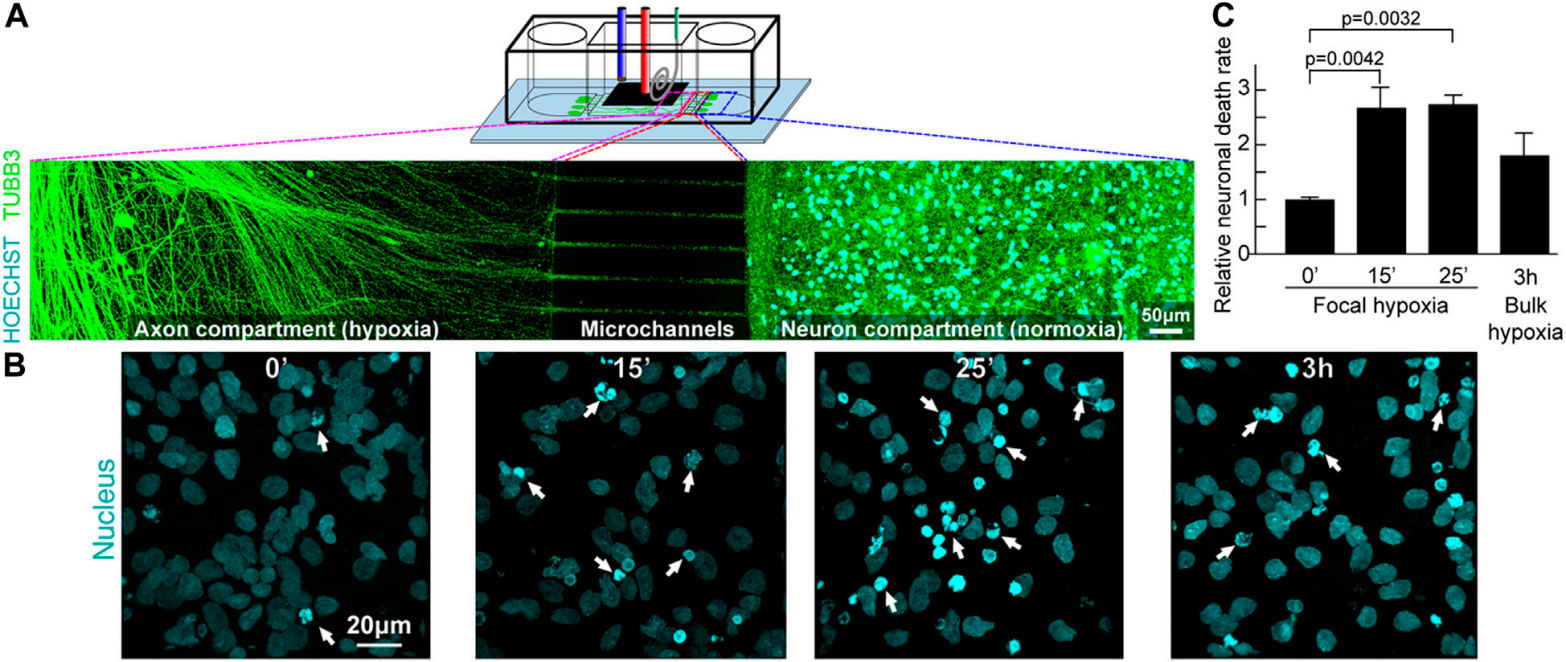
Focal hypoxia in a human cortical neuron microchannel model. **(A)** Schematic presentation of the microchannel model. Representative confocal tile image of neurons growing across the microchannels. Cyan: cell nucleus; green: axons. **(B)** Representative confocal image of cell nucleus (cyan) at the end chambers and axons in the central chamber after hypoxic insults. **(C)** Quantitative analysis of cells with chromatin condensation (n = 4).

The authors apologize for this error and state that this does not change the scientific conclusions of the article in any way. The original article has been updated.

