# Supplementary material for: Electrochemically induced in vitro focal hypoxia in human neurons
Source: Front Cell Dev Biol. 2022 Sep 28;10:968341. doi: 10.3389/fcell.2022.968341 (PMC9555746; doi:10.3389/fcell.2022.968341)
Supplement: Supplementary file 1 [file Presentation1.pdf]

Electrochemically induced in vitro focal hypoxia in human neurons

Joseph J. Y. Wong[1], Balazs V. Varga[2],
Ragnhildur Thóra Káradóttir[2] and Elizabeth A. H. Hall[1*]

[1]Department of Chemical Engineering and Biotechnology, University of Cambridge, Cambridge, United Kingdom. [2]Wellcome—MRC Cambridge Stem Cell Institute, Cambridge, United Kingdom

Supporting Information

Materials and Methods

1. Pt/C electrode construction

A copper wire was connected through a small hole punched at the centre of the electrode, sealed with silver epoxy overnight, followed by a top insulating layer of epoxy. A small rectangular indentation of about 2mm^2 was cut for placing the reference electrode close to the working electrode surface.

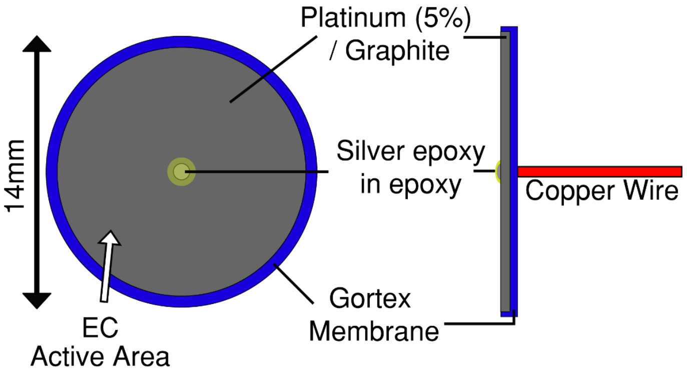

Schematic diagram of the construction of the platinum / graphite electrode.

2. Electrode array PCB construction

Gold wires were coiled around a 3D block with ridges to retrain the wire movement. The second block was bound to the gold-coiled block with the resin. The assembly was placed in a UV box for 3 hours at 60°C to allow crosslinking between the two pieces. The exposed wires were trimmed and soldered to the PCB. The PCB was designed with 16 contact points / side, making a total of 32 connections. The PCB was attached to an overlaying resin by a 3mm bolt and nut. The gold wire channels were filled with UV-curing resin and placed in the UV box for a total of 8 hours at 60°C in which the assembly was flipped every 2 hours. The final product was slotted into a 36-pin PCB edge connector. Silver epoxy was added to each PCB-pin connection to reduce contact resistance and only one side was used for the measurements.

3. PDMS culturing well construction

PDMS and curing agent (10:1) was cured at 80°C on a hot plate for 2 hours and placed in room temperature overnight before using. The Pt/C electrode covered most of the cross-sectional area. The scavenging efficiency was first studied by varying the position of the Pt/C electrode within the PDMS cell while keeping the depth of the solution constant at 5mm.

3. H_2O_2 enzymatic detection assay

H_2O_2 concentration was measured with an enzyme catalysed assay with horseradish peroxidase) as follow:

$$H_2O_2 + AUR \xrightarrow{HRP} Resorufin + H_2O \quad (4)$$

HRP catalyses the H_2O_2 and Amplex UltraRed (AUR) reaction to produce resorufin. Resorufin was excited by light of 490nm wavelength and a standard curve of emission at 592nm wavelength was obtained (supp. fig. 3,4). The solution contains a final concentration of 50μM of AUR, 1U/mL HRP and 40μL of sample in the 100μL final mixture.

Supplementary figures

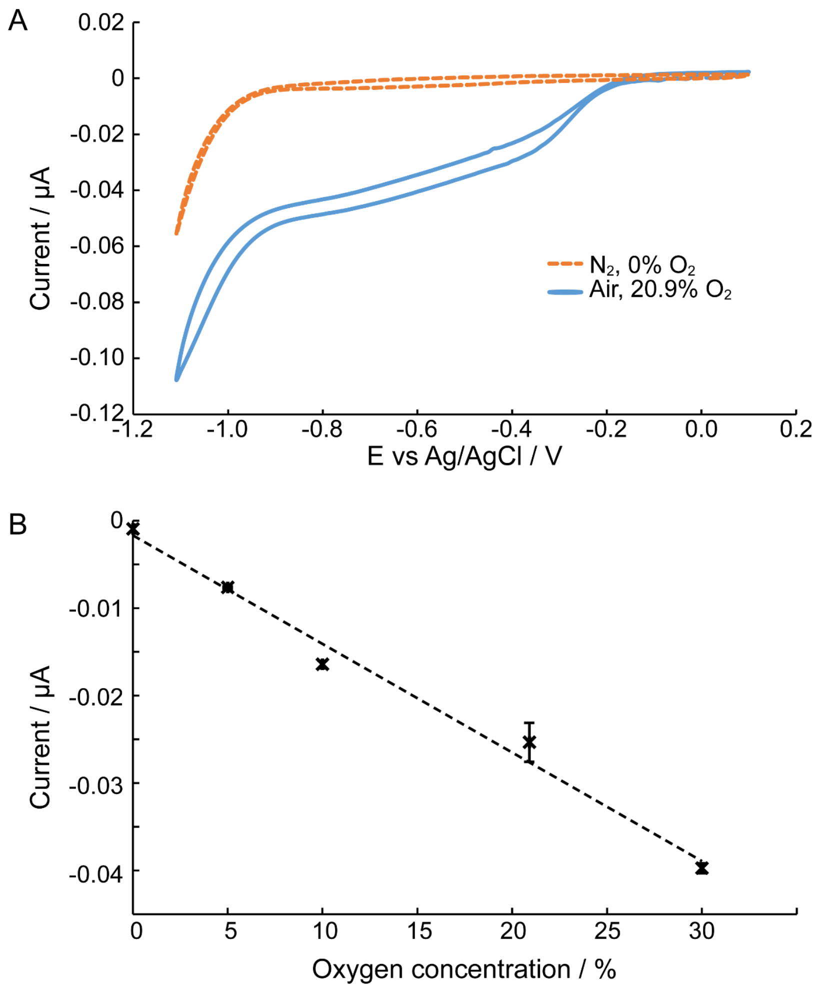

Figure S.1 Characterization of gold wire electrode for oxygen measurements. (A) Cyclic voltammetry of gold electrode (Ø:125μm) in degassed (orange) and aerated (blue) PBKCl. (B) Calibration curve of current recorded at gold electrode (-0.5V vs Ag/AgCl) against oxygen concentrations (n=3). Fitted curve: y = -0.00124x -0.00166.

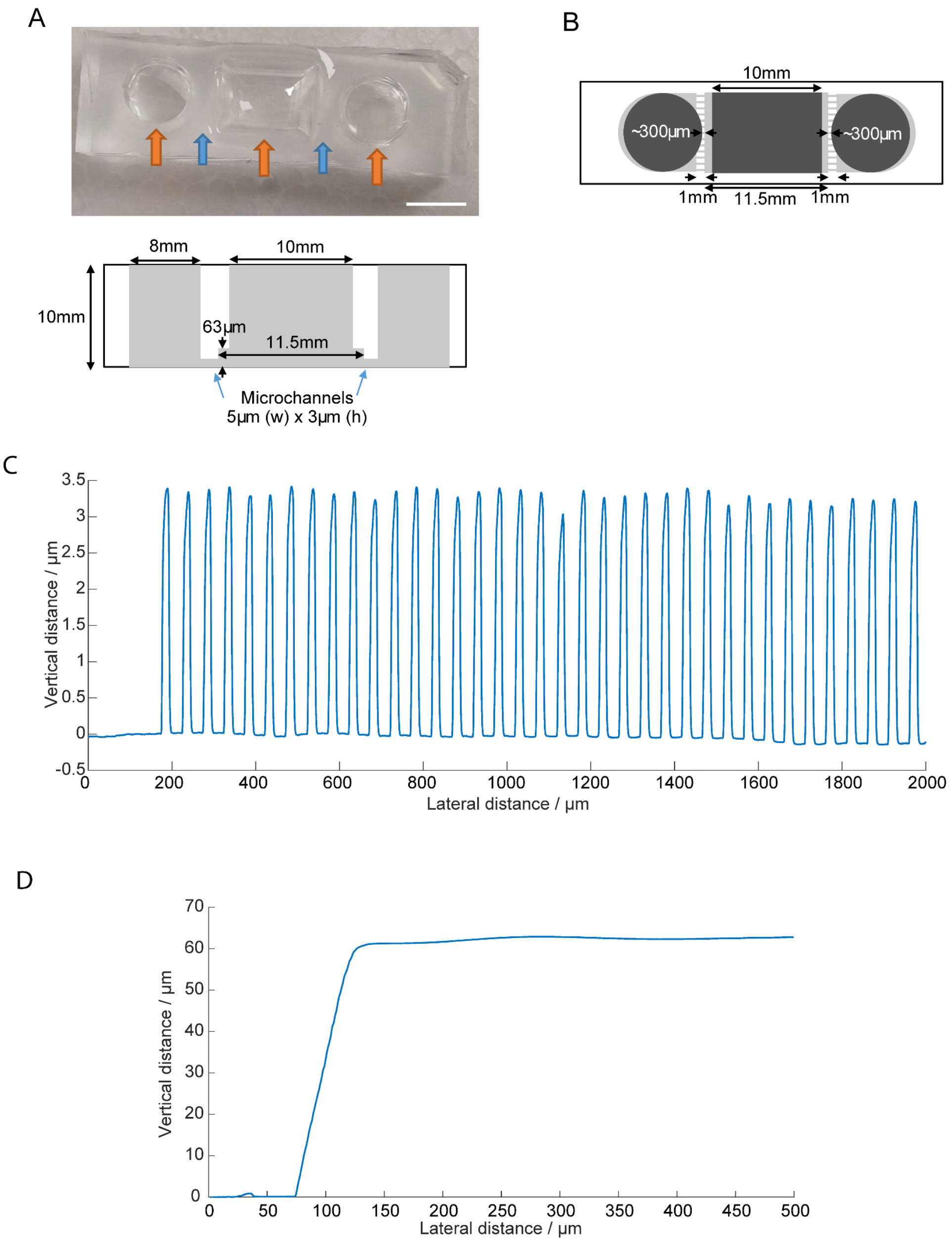

Figure S.2 Characterization of the microchannel system. (A) Photo image of the PDMS microchannel device. Blue arrows: microchannels; orange arrows: wells. Scale bar: 8mm. (B) Schematic diagram of the microchannel device and its dimensions. (C,D) Characterization of the microchannel device with the stylus profiler: first layer microchannels at 3.3 ± 0.1µm (C) and second layer culture wells before punching at 63 ± 1µm (D).

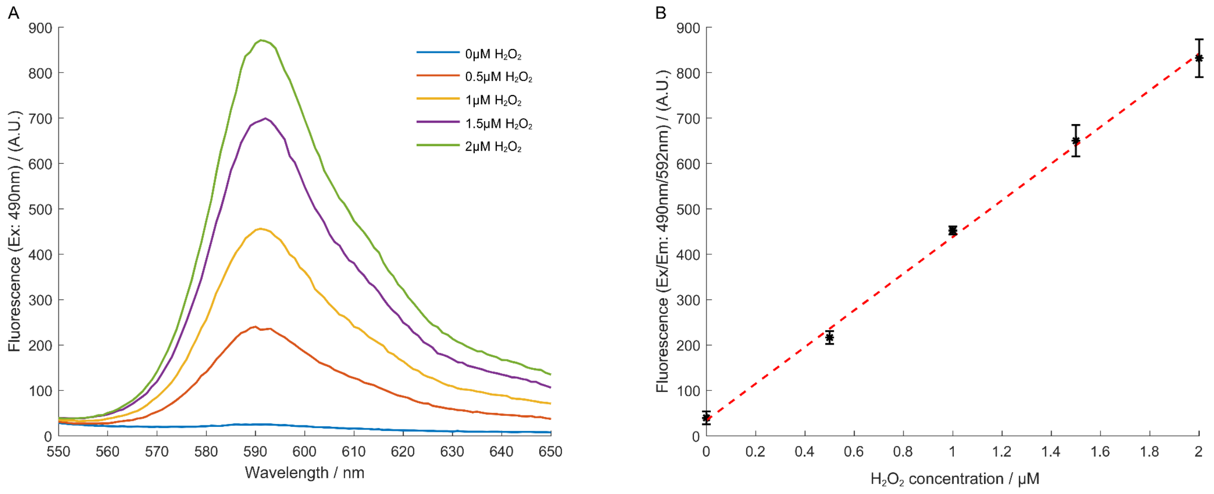

Figure S.3 Characterization of Amplex UltraRed for hydrogen peroxide measurements in PBKCl. (A) Fluorescence emission spectrum under excitation wavelength 490nm. (B) Calibration curve of Amplex UltraRed emission intensity against hydrogen peroxide concentrations (Ex.: 490nm; Em.: 592nm). The linear fit is y = 403.7x + 34.4.

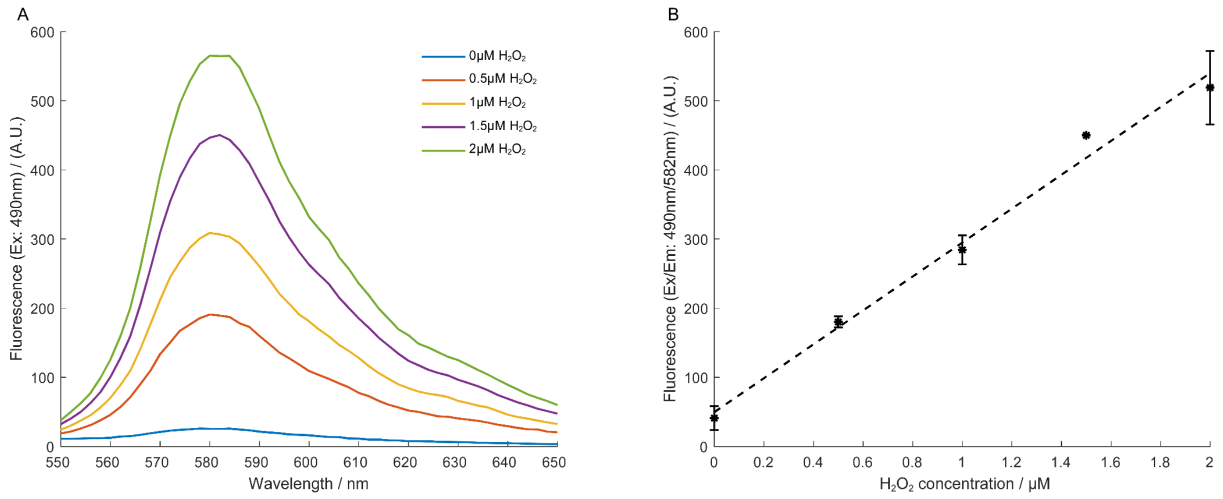

Figure S.4 Characterization of Amplex UltraRed for hydrogen peroxide measurements in culture medium. (A) Fluorescence emission spectrum under excitation wavelength 490nm. (B) Calibration curve of Amplex UltraRed emission intensity against hydrogen peroxide concentrations (Ex.: 490nm; Em.: 582nm). The linear fit is y = 245.2x + 49.7.

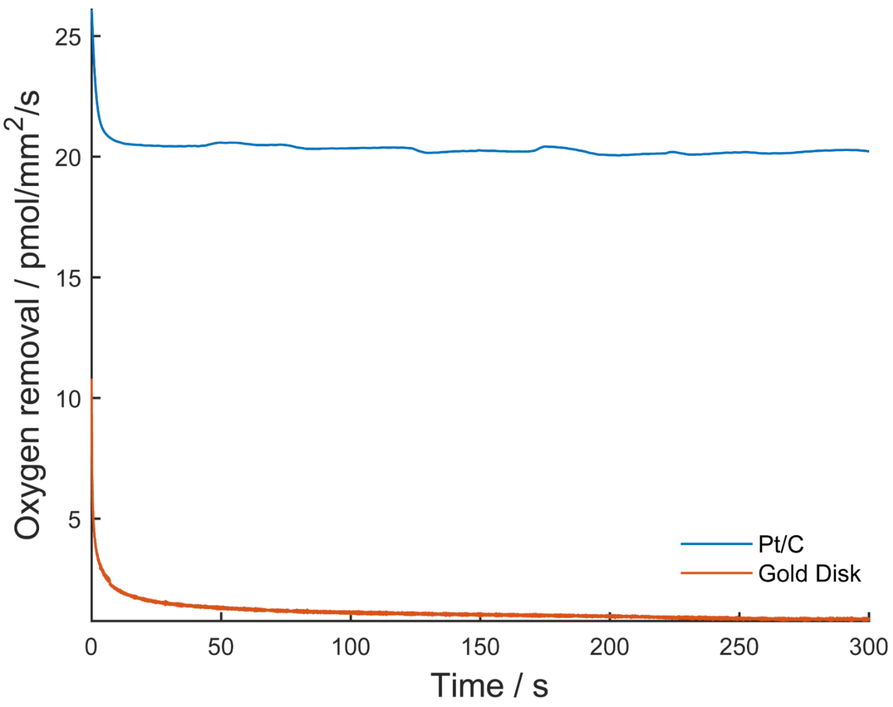

Figure S.5 Oxygen scavenging capability comparison of Pt/C and Au Disk in 0.1M KCl. Current density calculated from geometric surface area.

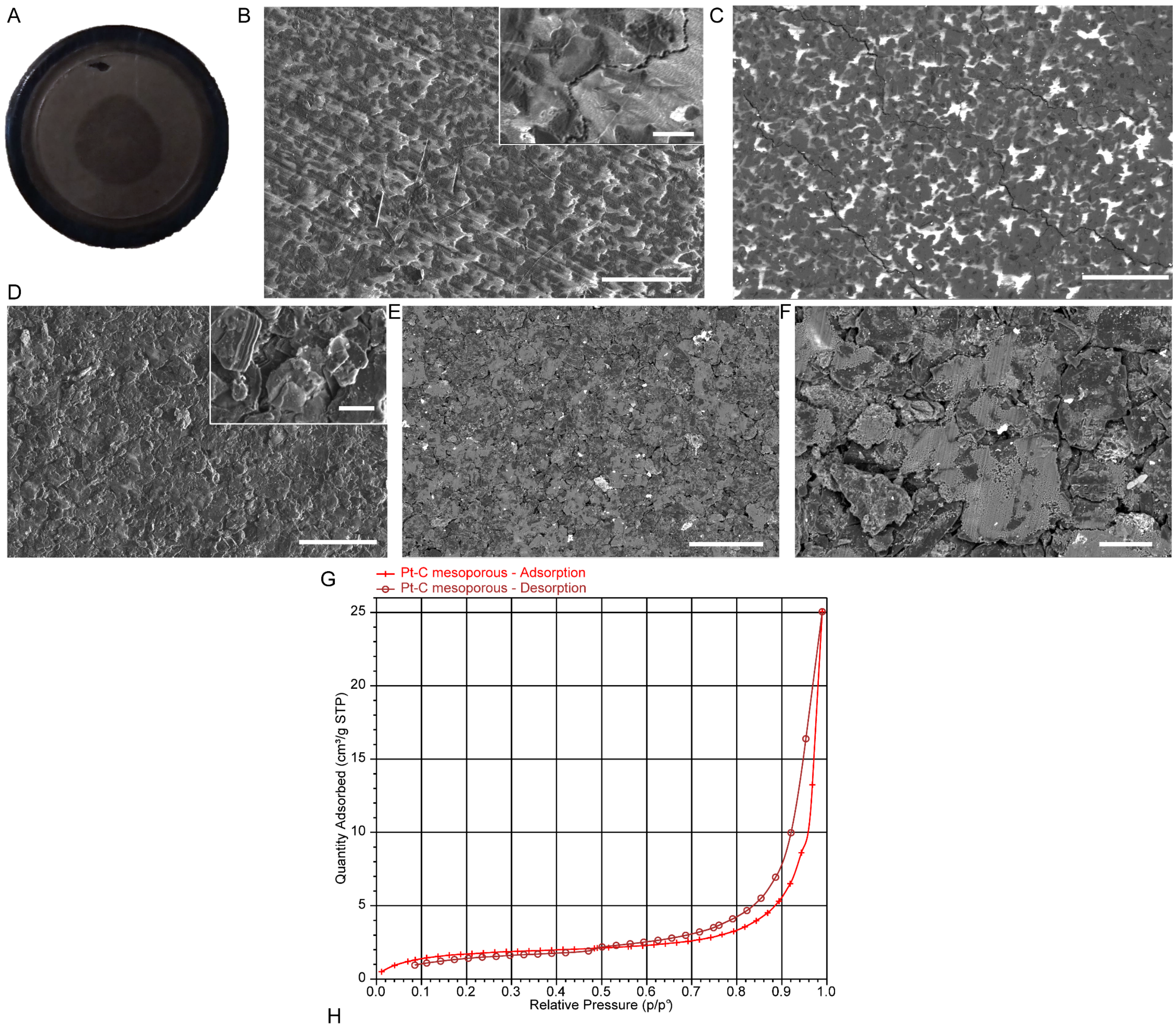

H

Sample (n=3)	Sample weight (mg)	Surface area (m²/g)
5% Pt/C	96.1	4.8 ± 0.7

Figure S.6 Pt/C surface analysis. (A) Camera image of the Pt/C material. Centre of the material has a duller surface than its surrounding. Dark blue rim is the gore membrane. (B,C) Representative SEM micrographs of the centre of Pt/C material. (B) Secondary electron micrograph at 500x magnification (scale bar: 50µm) and inset was at 5000x magnification (scale bar: 5µm). (C) Backscatter electron micrograph of the centre of the material (scale bar: 50µm). (D-F) Representative SEM micrographs of the dull silver colour region around the centre of Pt/C electrode. (D) Secondary electron micrograph at 500x magnification (scale bar: 50µm) and inset was at 5000x magnification (scale bar: 5µm). (E,F) Backscatter electron micrograph of the region around the centre. (E) Zoomed out micrograph (scale bar: 50µm). (F) Zoomed in micrograph (scale bar: 5µm). (G,H) BET curve and table for 5% Pt/C (n = 3).

The topography of the Pt/C electrode was investigated with the SEM (Figure S.6A-F). There were clear visual differences between the centre and the surrounding area of the electrode (Figure S.6A). This difference relates to the manufacturing process of the electrode. The centre had black colour in the SEM, whereas the surrounding had a dull silver surface. This is consistent with the sintering process, whereby the PTFE – Pt/C solution in an aluminium cup, is heated to near the melting point of PTFE; during the evaporation and sintering, material migrates from the centre and collects more towards the outside of the cup, also collecting slightly up the walls of the cup. It is then pressed onto Gore, producing an electrode with a thickness gradient from the outside to the centre (thinnest in the centre). SEM micrographs taken at the centre where the electrode is thinnest, showed a heterogenous layer with no obvious surface pores (Figure S.6B). There were also long parallel lines on the surface. These patterns are commonly seen on mechanically cut metallic surfaces and probably arise from the aluminium cup. When the material was pressed on the Gore membrane, the pattern may be carried over. Cracks were also observed on the surface with a wide dispersion of high intensity regions (Figure S.6C). The brighter regions are expected to be PTFE, which was used as the binding material, whereas the darker region would be graphite. This is supported by the publication by Kunz and Gruver[S1] (1975), in which they showed the electron photomicrograph of a microtomed platinum supported on Vulcan. The surrounding area where, during the evaporation and sintering process pushed material from the centre of the cup, showed a very different surface structure (Figure S.6D-F), rougher than that of the central region. Flakes of graphite were stacked in layers, analogous to SEM micrographs of ground graphite in the literature[S2-S4]. The higher magnification micrograph showed that pores smaller than 1μm in size were formed between the graphite flakes (Figure S.6D inset). It was more evident in the backscattered electron images that the pores exist in the region (Figure S.6E). It was also obvious that there were significantly less bright PTFE regions on the surface in this region, compared to the centre. This is consistent with the evaporation as the sintering begins, which leaves a higher deposition of PTFE in the centre. In the higher magnification backscattered electron image (Figure S.6F), a thin layer of high atomic weight particles was observed covering the graphite surfaces. According to a technical report by Prof. Michael Hitchman (personal communication), platinum aggregates dispersed on the material surface are expected, consistent with the observations of high atomic weight particles. It was evident that Pt/C had a heterogenous rough surface with small pores, that could be beneficial for electrochemical reaction.

S1. Kunz, H. R. & Gruver, G. A. The Catalytic Activity of Platinum Supported on Carbon for Electrochemical Oxygen Reduction in Phosphoric Acid. J. Electrochem. Soc. 122, 1279 (1975).

S2. Honbo, H., Takei, K., Ishii, Y. & Nishida, T. Electrochemical properties and Li deposition morphologies of surface modified graphite after grinding. J. Power Sources 189, 337–343 (2009).

S3. Ong, T. S. & Yang, H. Effect of atmosphere on the mechanical milling of natural graphite. Carbon 38, 2077–2085 (2000).

S4. Cvelbar, U., Pejovnik, S., Mozetiè, M. & Zalar, A. Increased surface roughness by oxygen plasma treatment of graphite/polymer composite. Appl. Surf. Sci. 210, 255–261 (2003).

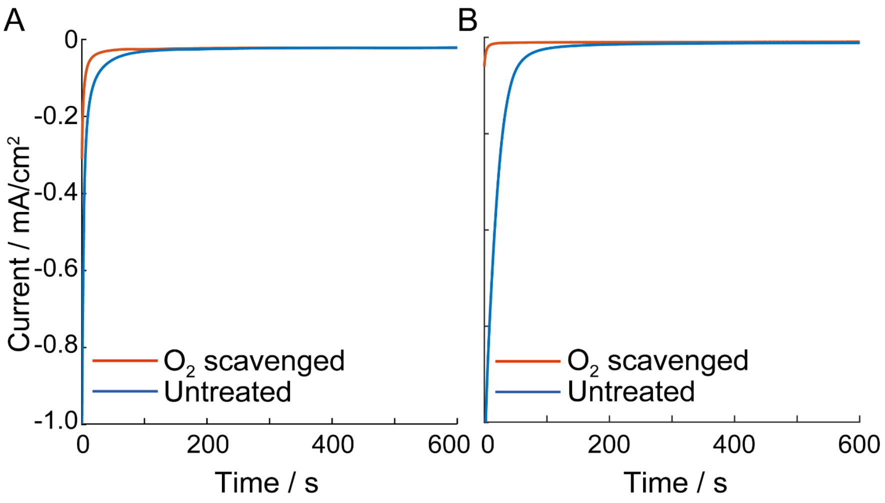

Figure S.7 Chronoamperometry of Pt/C electrode for oxygen adsorption. Pt/C polarized at -0.7V vs Ag/AgCl electrode in PBKCl measured in air (A) and nitrogen (B). O_2 scavenged Pt/C electrode (Red); Untreated Pt/C electrode (Blue).

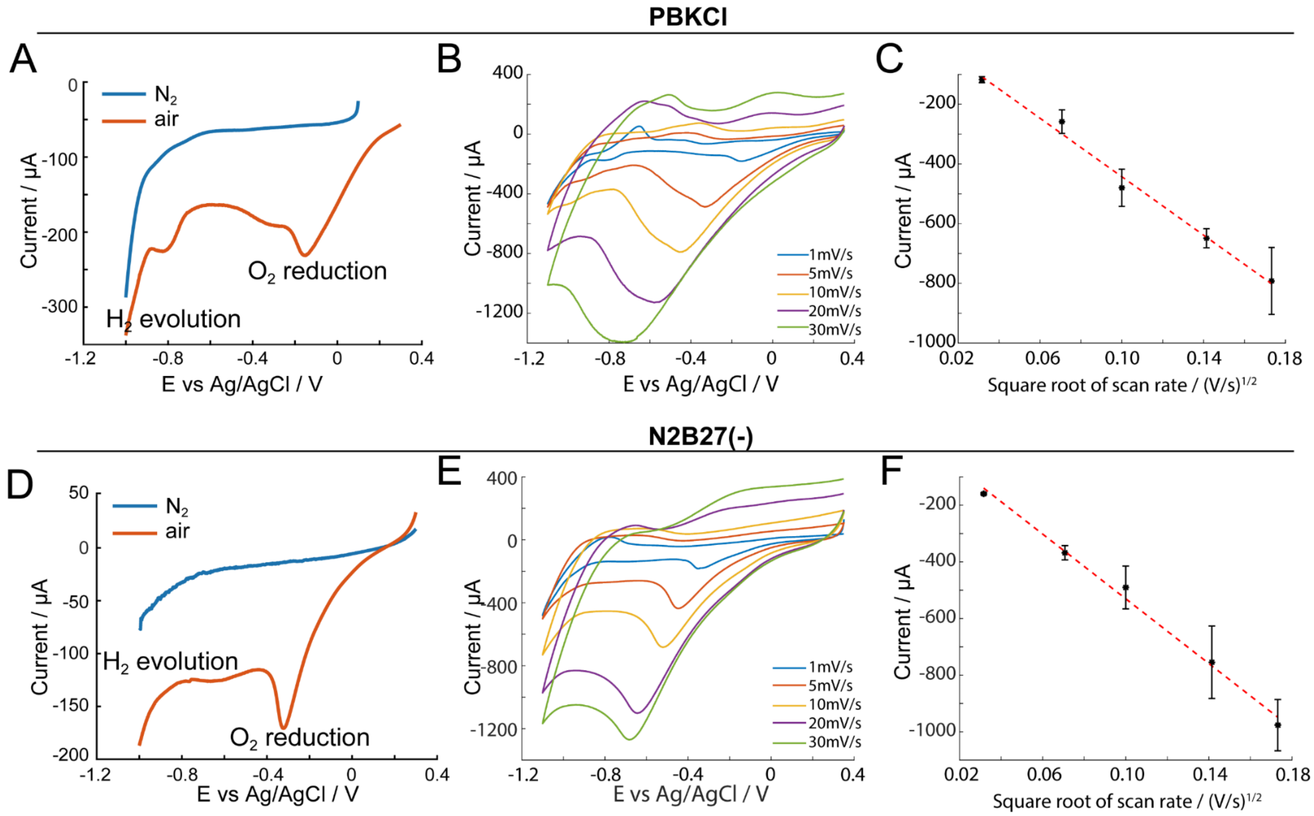

Figure S.8 Characterisation of Pt/C in PBKCl (A-C) and N2B27(-) (D-F). (A, D) Linear sweep voltammetry in air and nitrogen environment at 1mV/s. (B, E), Cyclic voltammetry with varying scan rate (1mV/s to 30mV/s) and (C, F) corresponding plot of peak current to square root of scan rate. Projected linear curve (red dotted line) is displayed on the image. The equations in (C) and (F) are $y = –5.71×10^3x + 40.9$ ($R^2 = 0.9893$) and $y = –4.89×10^3x + 46.9$ ($R^2 = 0.9929$), respectively. The calculated electrochemical active surface area were $5.97 cm^2$ and $5.19 cm^2$ for PBKCl and N2B27(–), respectively.

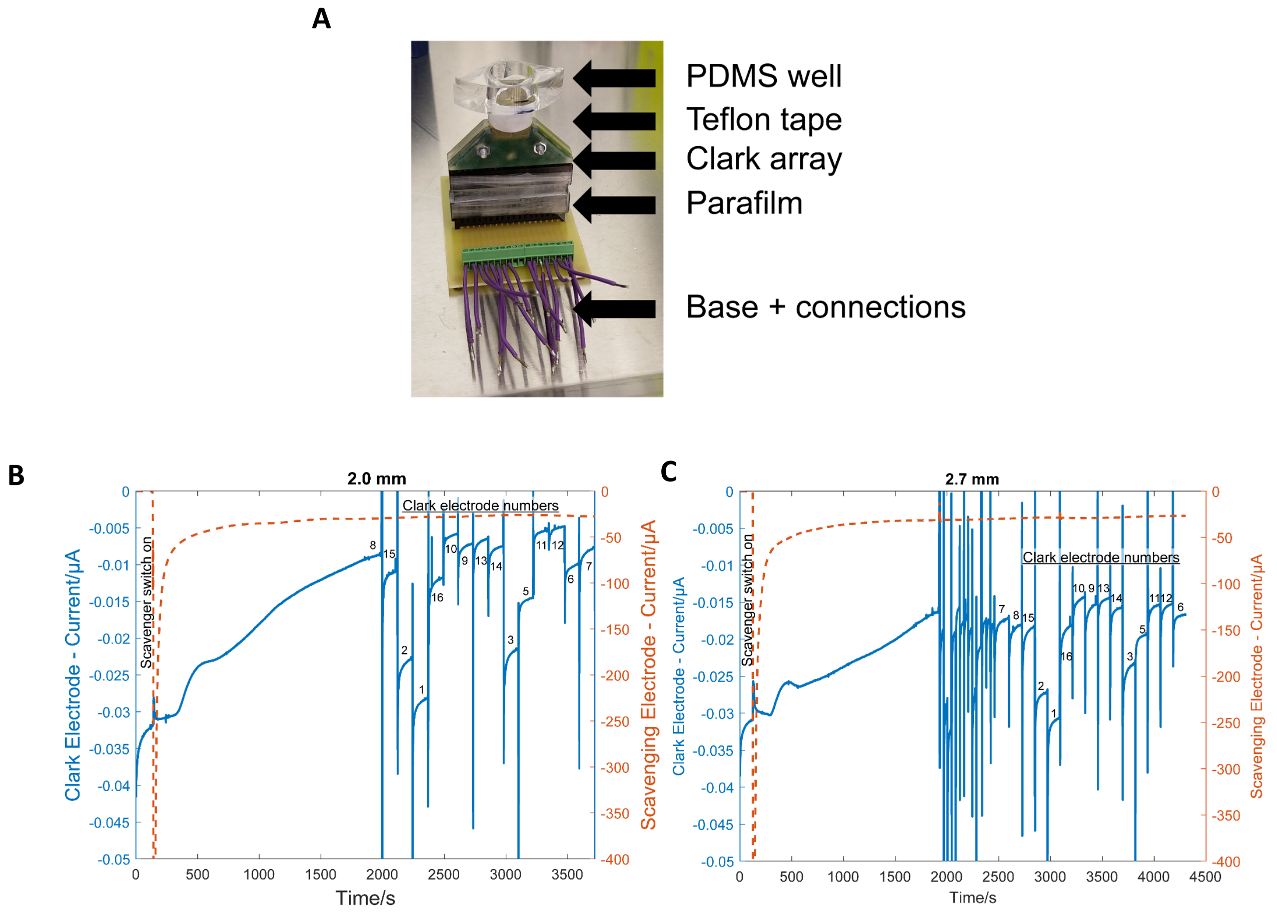

Figure S.9 Characterization of resultant oxygen concentration in focal hypoxia system. (A) Schematic diagram of the gold electrode array for oxygen characterization. (B,C) Oxygen concentration across the base under 2.0mm (B) or 2.7mm (C) height of scavenging electrode. Gold electrode stabilized for 2 min and then scavenging electrode is switched on. After 30 min, current at different positions were measured for 2 minutes each. In (C), 30 second measurements were conducted between 32min and 41min to test the responsiveness before the 2-minute measurements. The currents measured were translated to relative oxygen concentrations (Fig. 4) based on the calibration curve (Fig. S.1). Current spikes were due to noise when the leads were connected to the potentiostat.

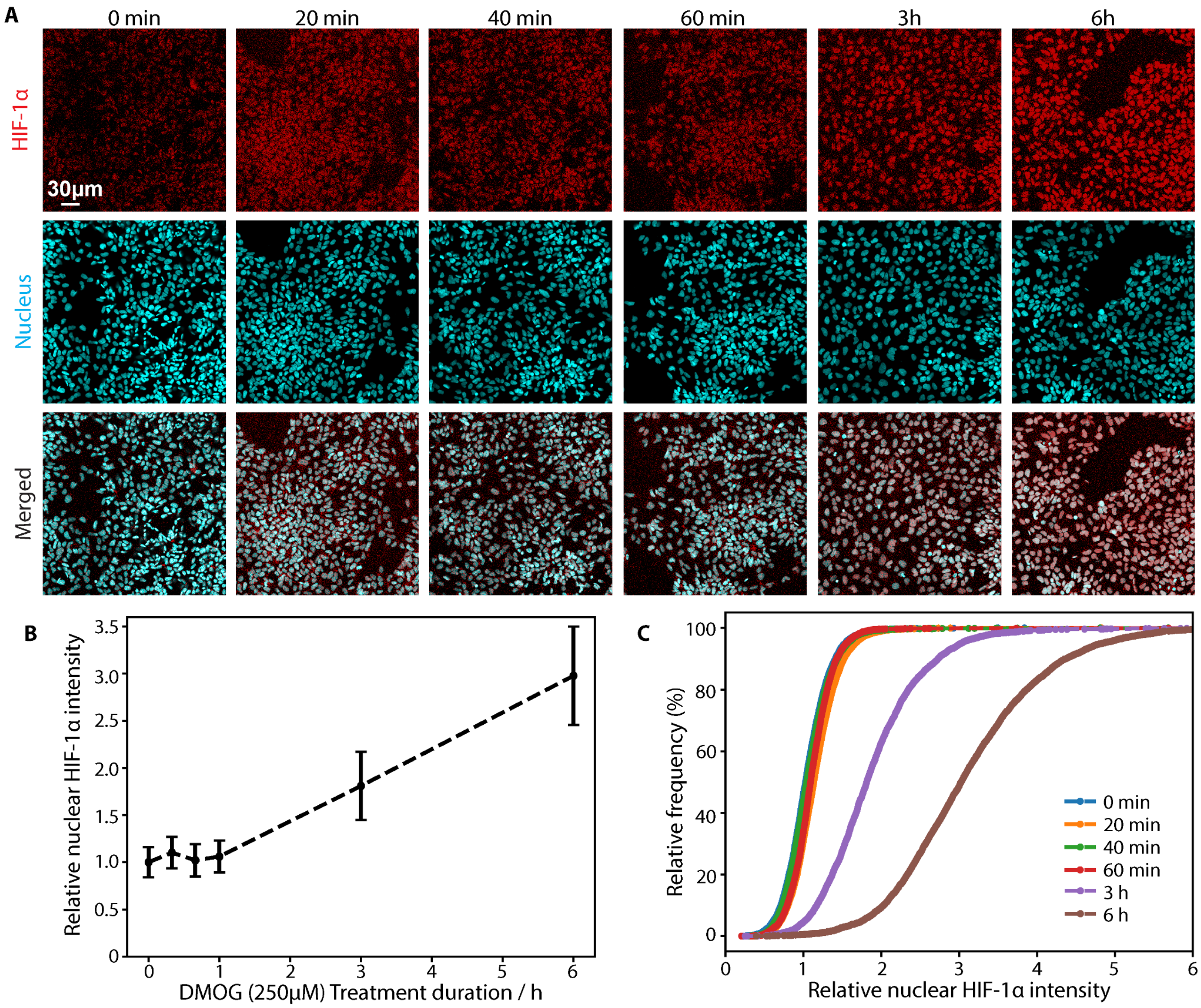

Figure S.10 Acute hypoxia of hNPC with DMOG 250μM (n = 3). (A) Representative confocal images of hNPCs under DMOG treatment. HIF-1α (red), nucleus (cyan). (B) Plot of the mean nuclear HIF-1α intensity under the DMOG treatment. (C) Frequency distribution analysis of the nuclear HIF-1α intensity under different durations. Intensity is normalized against the negative control.

Under the DMOG 250μM treatment, increase in HIF-1α intensity was only observed after 3 hours. Negligible differences were observed for the first 60 minutes of treatment. Comparing with that observed in figures 5 and 6 of the main text, environmental change of oxygen by eLOS is more efficient in generating acute hypoxia in hNPCs than with chemically-mimicked hypoxia.
